# Characterization, genome analysis and genetic tractability studies of a new nanocellulose producing *Komagataeibacter intermedius* isolate

**DOI:** 10.1038/s41598-022-24735-z

**Published:** 2022-11-28

**Authors:** Pietro Cannazza, Antti J. Rissanen, Essi Sarlin, Dieval Guizelini, Carlotta Minardi, Pauli Losoi, Francesco Molinari, Diego Romano, Rahul Mangayil

**Affiliations:** 1 Department of Food, Environmental and Nutritional Sciences (DeFENS), University of Milan, Via Celoria 2, 20133 Milan, Italy; 2 Faculty of Engineering and Natural Sciences, Tampere University, Tampere, Finland; 3 Graduate Program in Bioinformatics, Sector of Professional and Technological Education, Federal University of Parana (UFPR), Curitiba, PR Brazil; 4 Department of Bioproducts and Biosystems, Aalto University, Espoo, Finland

**Keywords:** Applied microbiology, Bacterial genetics, Bacterial synthetic biology, Bacterial genomics, Biopolymers, Bacterial genes

## Abstract

Bacterial nanocellulose (BC) is a highly versatile biopolymer currently pursued as a material of choice in varied themes of biomedical and material science research fields. With the aim to extend the biotechnological applications, the genetic tractability of the BC producers within the *Komagataeibacter* genus and its potential as an alternative host chassis in synthetic biology have been extensively studied. However, such studies have been largely focused on the model *Komagataeibacter* spp. Here, we present a novel *K. intermedius* strain capable of utilizing glucose, and glycerol sources for biomass and BC synthesis. Genome assembly identified one bacterial cellulose synthetase (*bcs*) operon containing the complete gene set encoding the BC biogenesis machinery (*bcsI*) and three additional copies (*bcsII–IV*). Investigations on the genetic tractability confirmed plasmid transformation, propagation of vectors with pBBR1 and p15A origin of replications and constitutive and inducible induction of recombinant protein in *K. intermedius* ENS15. This study provides the first report on the genetic tractability of *K. intermedius*, serving as starting point towards future genetic engineering of this strain.

## Introduction

Bacterial nanocellulose (BC) is a versatile biopolymer predominantly synthesized by species of *Acetobacteraceae* family and is widely studied in *Komagataeibacter* genera. In *Komagataeibacter* spp., BC synthesis is controlled by cyclic dimeric guanosine monophosphate (c-di-GMP) and occurs via the catalytic activity of bacterial cellulose synthetase (*bcs*) operon encoding BcsABCD proteins^1^. Briefly, the process involves c-di-GMP activation of BcsAB and formation of glucan units employing UDP-glucose, generated via the enzymatic activities of glucokinase, phosphoglucomutase and UDP-glucose pyrophosphorylase. The glucan units are then crystallized into nanocellulose fibrils by BcsD and secreted through the outer membrane pore formed by BcsC, which is later bundled extracellularly to form BC pellicle^2^. Unlike plant-based cellulose, BC offers unique properties such as high purity (i.e., devoid of lignin, hemicellulose, or pectin), biocompatibility, high water retention, superior mechanical and optical properties which has been utilized commercially in medical wound-dressings, health foods and presently researched as a sustainable material in electrical, sensing and biomedical applications^2^.

Among the *Komagataeibacter* spp., *K. xylinus*, *K. rhaeticus* and *K. hansenii* are considered as the model organisms for studies on nanocellulose production^3,4^. Despite the bioprocess and engineering advancements in the model organisms^5,6^, low production titers have encouraged the researchers to isolate novel nanocellulose producing bacteria^7–10^. Although the studies include whole genome analysis and characterization of the isolates’ capacity to valorize agro- and industrial waste effluents for BC biogenesis^9,11^, studies on the genetic tractability of *Komagataeibacter* spp. have been largely constrained to the model nanocellulose producers^6^. Here we report the isolation and characterization of a novel *K. intermedius* isolate from Kombucha SCOBY (symbiotic culture of bacteria and yeast). Genome sequencing and whole-genome analysis identified new genes associated with nanocellulose synthesis and putative genes involved in pentose bioconversion. Furthermore, the work studied the applicability of transformation protocols established for related *Komagataeibacter* spp. and identified suitable plasmid backbones, constitutive and inducible promoter systems.

## Materials and methods

### Chemicals and materials

Kombucha SCOBY, chemicals, GeneJET Genomic DNA Purification Kit and crude glycerol was in this study were obtained from sources mentioned in Mangayil et al.^9^.

### Isolation, characterization, and culturing of BC-producing strains

Isolation and biochemical characterization of the bacterial cellulose producing isolate was conducted as described in Mangayil et al.^9^. For pre-inoculum preparation, 10 µl from the glycerol stock were inoculated into 5 ml HS-glucose medium (g/L, 5 peptone, 5 yeast extract, 2.7 Na_2_HPO_4_, 1.15 citric acid and 20 g/L glucose) and incubated statically for 5 days at 30 °C. The BC pellicles were lysed overnight (O/N) with 1% cellulase and the released cells washed thrice with 1X Phosphate Buffered Saline (PBS; g/L, g/L, 8 NaCl, 0.2 KCl, 1.44 Na_2_HPO_4_, and 0.24 KH_2_PO_4_; pH 7.4) were used as the pre-inoculum in subsequent experiments. The BC production experiments were conducted through static cultivations in 6-well culture plates (Argos Technologies, Cole-Parmer, US) containing HS medium individually supplemented with 20 g/L of studied carbon sources^9^.

### BC film preparation and material characterization

BC film preparation and dry weight measurements were conducted as mentioned in Mangayil et al.^12^. Structural and chemical properties of the BC sheets synthesized from glucose, pure glycerol and crude glycerol were analysed using a scanning electron microscope (SEM; Zeiss ULTRAPlus, Germany) and X-ray diffractometer (XRD; Empyrean multipurpose diffractometer, PANalytical B.V, US), respectively, as described in Mangayil^12^. The thermal properties were analysed under N_2_ atmosphere using a Thermogravimetric analyser (TGA; TG 209 F3 Tarsus, Netzsch-Gerätebau GmbH, Germany^9^).

### Identification and phylogenetic classification of the BC producing bacterium

For strain identification, the isolate was statically cultured in loosely capped 50 ml Corning tubes at 30 °C in 10 ml HS-glucose medium for 5 days. The BC pellicle was lysed, and the cells were washed as previously described. The genomic DNA (gDNA) was extracted using the GeneJET Genomic DNA Purification Kit (Thermo Scientific, USA) as per the manufacturer’s instructions. The 16S rRNA gene sequence amplified using the identification service from Macrogen (primer pairs 27F, 5′-AGAGTTTGATCMTGGCTCAG-3′ and 1492R, 5′-TACGGYTACCTTGTTACGACTT-3′) and sequencing (primer pairs 785F, 5′-GGATTAGATACCCTGGTA-3′ and 907R, 5′-CCGTCAATTCMTTTRAGTTT-3′) can be found in the NCBI GenBank database under the accession number MT094082. Homology comparisons of the 16S rRNA gene were conducted using the nucleotide BLAST (blastn)^13^ tool against the NCBI GenBank 16S rRNA gene sequence repository for *Komagataeibacter* (taxid:1434011). Using the MEGA X^14^ integrated tool, alignments against the 16S rRNA gene sequences of *Komagataeibacter* type strains were conducted using ClustalW^15^ and the evolutionary relationship was inferred using the Neighbor-Joining method^16^ with Kimura 2-parameter model^17^ and bootstrapping (1000 times) at uniform rates.

### Genome sequencing, assembly and bioinformatics

*K. intermedius* ENS15 gDNA was sequenced at Novogene Europe (Cambridge, UK) using the Illumina Novaseq 6000 system. The raw reads were quality controlled using FastQC^18^, trimmed using Trimmomatic^19^ and de-novo assembled using SPAdes^20^. The misassembles were identified using QUAST (5.0.2)^21^. Contig reordering, missassembly correction and gap filling was conducted with GFinisher^22^ tool using *K. intermedius* AF2 genome (GenBank accession: GCA_000817255.2) as the reference. The scaffolds were manually linked, the genome completeness was analysed by CheckM tool^23^, and annotated using Prokka^24^. Phylogenetic relatedness and genome-based taxonomy was performed in Type Strain Genome Server (TYGS)^25^ (https://tygs.dsmz.de/, accessed on 23.07.2021). The plasmid sequences in the raw reads and draft genome were identified using plasmidSPAdes^26^ and Recycler^27^, respectively. The genome assembly can be found in the NCBI GenBank under the accession number GCA_000964425.1.

The genes encoding the Bcs machinery, extracellular matrix formation, and carbohydrate uptake and catabolism were identified through manual search within the Prokka annotated GenBank file using Unipro UGENE software (v. 33.0)^28^ and confirmed using protein BLAST (blastP)^13^ against the NCBI non-redundant database. The phylogenetic relatedness and amino acid identities of *bcs* operon proteins, BcsAB and BcsC, were identified using MUSCLE using the default settings^29^. The phylogenetic tree was built in MEGA X integrated tool^14^ using the Neighbour-Joining method and the evolutionary distances were computed using the Poisson correction method^30^. The domain architecture predictions were conducted through the NCBI conserved domain database (NCBI CDD)^31^ and InterproScan^32^ searches.

### Preparation of electrocompetent *K. intermedius* ENS15 cells and plasmid transformation

The electrocompetent cells were prepared as described by Mangayil et al.^12^. To determine the appropriate antibiotic concentration for plasmid transformation, single aliquots (100 µl) of *K. intermedius* ENS15 competent cells were plated, in triplicates, on to HS-glucose agar supplemented with kanamycin (Kan; 400 mg/L, 500 mg/L, 600 mg/L, 700 mg/L) and chloramphenicol (Cm; 200 mg/L, 300 mg/L, 340 mg/L, 400 mg/L, 500 mg/L, 550 mg/L, 600 mg/L, 700 mg/L). The colony forming units (CFUs) were calculated after six days of static incubation at 30 °C. A blank cultivation, HS-glucose agar plates devoid of antibiotic supplementation and cells, was included as control.

Plasmids with seven different origins of replication (Table S1) obtained from Standard European Vector Architecture (SEVA) repository (http://seva-plasmids.com/) were tested for its replication in *K. intermedius* ENS15. As kanamycin was identified as an unsuitable selection marker, the native antibiotic resistance gene in the SEVA vectors with kanamycin resistance gene (pSEVA211, pSEVA241, pSEVA261, pSEVA271, pSEVA281 and pSEVA291) were replaced with chloramphenicol resistance (CmR) gene from pSEVA331 via SmiI/BoxI restriction and one-step cold phosphorylation-ligation using T4 poly nucleotide kinase and T4 DNA ligase. The resulting plasmids, named according to the SEVA guidelines as pSEVA311, pSEVA341, pSEVA361, pSEVA371, pSEVA381 and pSEVA391, were verified using PCR [PS5_SEVA and PS6_SEVA (targeting the origin of replication) and Fwd_CmR and Rev_CmR (targeting the CmR gene) primers (Table S1)] and sequencing.

Plasmid DNA (~ 200 ng) mixed with 100 µl of electrocompetent *K. intermedius* ENS15 cells were transferred to 1 mm electroporation cuvettes, pulsed (MicroPulser, BioRad) at 2.5 kV and immediately recovered with 1 ml of prewarmed HS medium containing 5% cellulase. The recovered cells were transferred to 15 ml culture tubes and incubated O/N at 30 °C and 220 rpm. After the incubation period, the transformants were plated on individual HS-glucose agar plates containing appropriate antibiotic concentrations and grown statically at 30 °C for 5–7 days. The transformation efficiency (%) was calculated by dividing CFU with the transformed DNA amount.

### Characterization of constitutive promoters

To identify a suitable promoter for constitutive gene expression in *K. intermedius* ENS15, pSB1C3 vector harboring eight constitutive promoters (J23104, J23105, J23106, J23107, J23100, J23111, J23113, J23116, J23118) were selected from the *E. coli* Anderson promoter collection (iGEM Parts Registry, Spring 2017 Distribution). Promoters J23104 and J23105 that have been previously demonstrated to cater the strongest and weakest gene expression, respectively, in *Komagataeibacter* spp. were included as experimental controls^4,6^. To confirm plasmid replication and relatable gene expression, the genetic elements (promoter, RBS, mRFP1 and double terminator) in pSB1C3 were restricted with XbaI/PstI and ligated to similarly digested pSEVA331 vector. The resulting plasmids (Table S1) were transformed into *K. intermedius* ENS15 and plated onto HS-glucose agar containing 320 mg/L Cm and 1% v/v cellulase. Single colonies were individually picked and cultivated in 5 mL HS-Glu containing 32 µg/ml Cm and 1% v/v cellulase at 30 °C at 300 rpm for 3 days. Cells were collected (8000 g for 5 min), washed twice with 1X PBS and resuspended in 1 ml PBS. The promoter strength was evaluated by analysing the mRFP1 fluorescence, normalized per unit optical density at wavelength 600 nm (OD_600nm_), using Spark Multimode Microplate Reader (TECAN, Switzerland) at excitation wavelength 580 nm and emission wavelength 610 nm. For constitutive mRFP1 expression in pellicles, 50 µL of the recombinant *K. intermedius* cells harbouring the selected genetic elements were inoculated in 6-well culture plates and statically grown in 10 ml of HS-glucose medium containing 32 mg/L CmR at 30 °C for 6 days.

### Characterization of inducible gene expression constructs

The suitability of pLux-LuxR (Addgene #78281) and pTet-TetR (Addgene #78283), inducible systems that has been reported to function in *K. xylinus* and *K. rhaeticus*^4,6^ strains were tested for its functionality in *K. intermedius* ENS15. Furthermore, mRFP1 gene induction via a Cumate inducible system (Addgene #119872) was studied in *K. intermedius* ENS15^33^. To ensure functionality of the Cumate inducible system in *K. intermedius* ENS15, the induction cassette (comprising of CuO/CymR, RBS and sfGFP) was amplified using EcoRI_Cumate and xbaI_Cumate primers from pCT5-bac2.0 (Table S2), restricted with EcoRI/XbaI enzymes and ligated with similarly digested pSEVA331 vector, resulting in pSEVA331Cumate_sfGFP plasmid. To avoid any discrepancy due to varying reporter gene and RBS among the studied vectors, the sfGFP and RBS in pSEVA331Cumate_sfGFP plasmid was replaced with the RBS and mRFP1 from pSEVA J23104 mRFP1. The pSEVA 331_Cumate_sfGFP plasmid backbone (amplified using Vector.FOR_Cumate and Vector.REV_Cumate primers to exclude the RBS and sfGFP gene) was assembled with the PCR product (containing RBS and mRFP1 from J23104-mRFP1-331Bb) amplified using Fragment.FOR _Cumate and Fragment.REV _Cumate primers via NEBuilder HiFi DNA Assembly Master Mix, resulting in pCumate plasmid. Plasmid transformation, propagation of recombinant *K. intermedius* ENS15 cells and fluorescence tests were conducted as described in Section “Characterization of constitutive promoters”.

## Result and discussion

### Isolation, characterization and classification of BC-producing strain

**Figure 1:**
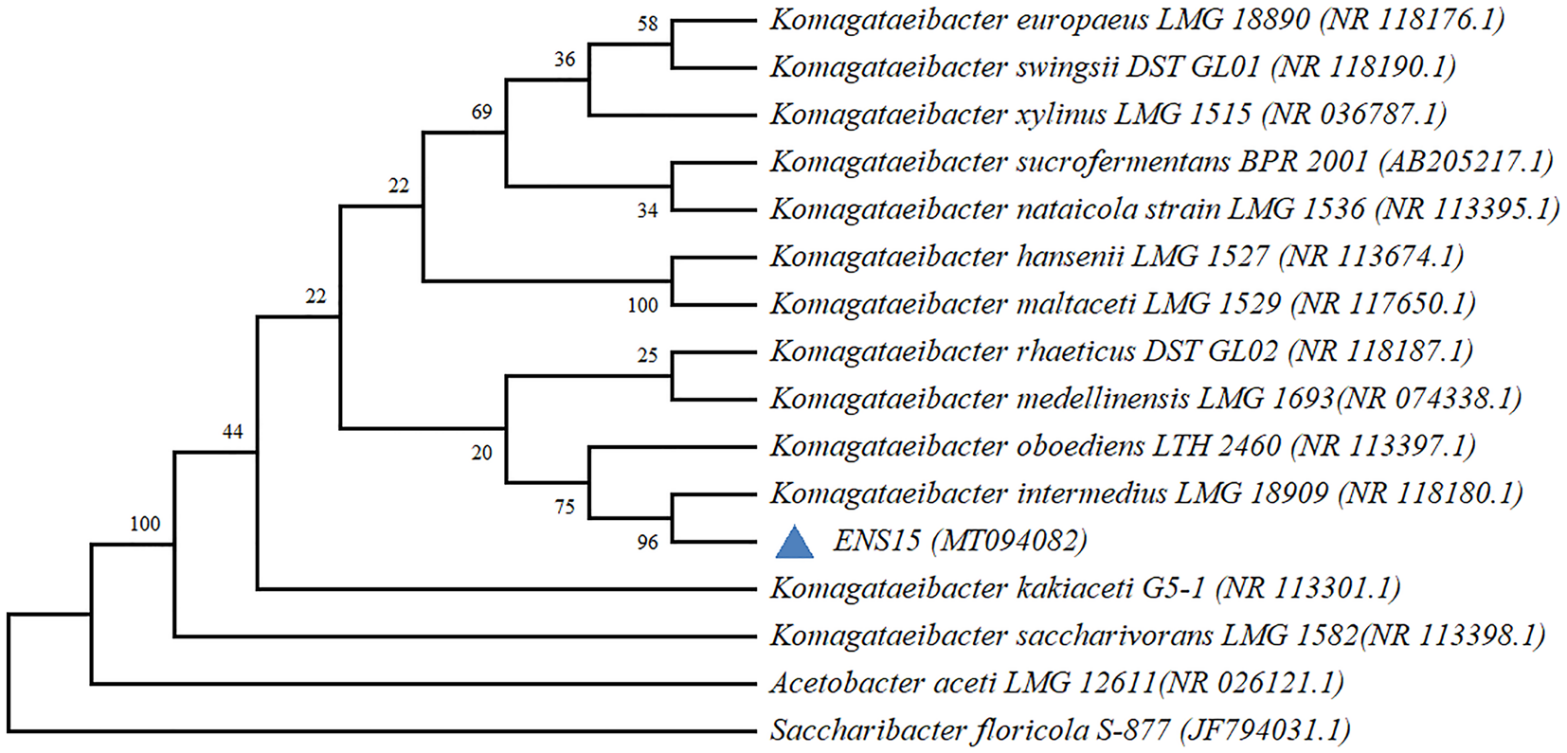
16S rRNA gene-based phylogenetic relationship of ENS15 and *Komagataeibacter* type strains (NCBI accession numbers in parenthesis). The tree, drawn using MEGA X integrated tool^14^ (https://www.megasoftware.net/, version 10.2.4), was rooted using *Saccharibacter floricola* JCM 12116. The percentage of replicate trees in which the associated taxa clustered together in the bootstrap test (1000 replicates) are shown next to the branches.

Isolation of single clones from CaCO_3_ halo zones in Glucose-Yeast Extract-Calcium carbonate agar and iterated subculturing in HS-Glu agar resulted in enrichment of an isolate with beige-coloured, smooth-edged and umbonate shaped colonies characteristics (Fig. S1A). The isolate is hereafter called ‘ENS15’. Under 100X magnification, the isolate appeared as rod-shaped cells either singularly, in pairs or in chains, having cell sizes in the range of 2.6–5.0 µm *0.6–0.7 µm (Fig. S1B). Phylogenetic positioning with the 16S rRNA gene sequences of *Komagataeibacter* type strains affiliated the isolate to *K. intermedius* LMG 18909^ T^ (Fig. 1).

The ENS15 strain was able to grow in MA/9 medium supplemented with 30% glucose but grew poorly when cultured in medium containing 3% ethanol, 0.35% acetic acid, and 3% ethanol and 4% acetic acid as the sole carbon sources. Therefore, the remaining studies were conducted in PY medium (Fig. S2). The isolate did not require acetic acid for growth, demonstrated acetate and lactate oxidization, and acetic acid overoxidation (Fig. S3).

### Whole-genome analysis of *K. intermedius* ENS15

To gain valuable insights on carbohydrate uptake and metabolism and BC biogenesis machinery, *K. intermedius* ENS15 genome was sequenced to a coverage of 400X and assembled de novo using SPAdes. Due to the low genome quality of the *K. intermedius* type strain LMG 18909^ T^ (Strain TF2, GenBank assembly accession: GCA_000964425.1), scaffolding with GFinisher was conducted using *K. intermedius* AF2 genome (GenBank assembly accession: GCA_000817255.2) as the reference. The assembly statistics are presented in Table S3.

To confirm the strain’s phylogenetic positioning among the *Komagataeibacter* sp., a genome-based validation using the TYGS database was conducted. The server calculates the digital DNA-DNA hybridization (dDDH) values between the query and subject genomes based on three Genome BLAST distance phylogeny formulas: Genome to genome distance calculator formula 1 (GGDH; d_0_) calculates the length of all high-scoring segment pair (HSP) divided by total genome length, GGDH formula 2 (d_4_) calculates the sum of all identities found in HSPs divided by overall HSP length and GGDH formula 3 (d_6_) calculates the sum of all identities found in HSPs divided by total genome length. As the formula d_4_ is independent of genome length and thus robust against the use of incomplete draft, it is used for validation. The pairwise comparison results confirmed the isolate’s affiliation with *K. intermedius* type strain LMG 18909^ T^ and *K. intermedius* AF2 with a digital DNA-DNA hybridization (distance formula *d*_*4*_*;* sequence level similarity; subspecies clustering threshold of 70%) score of 99% and 97%, respectively (Table S4).

**Figure 2:**
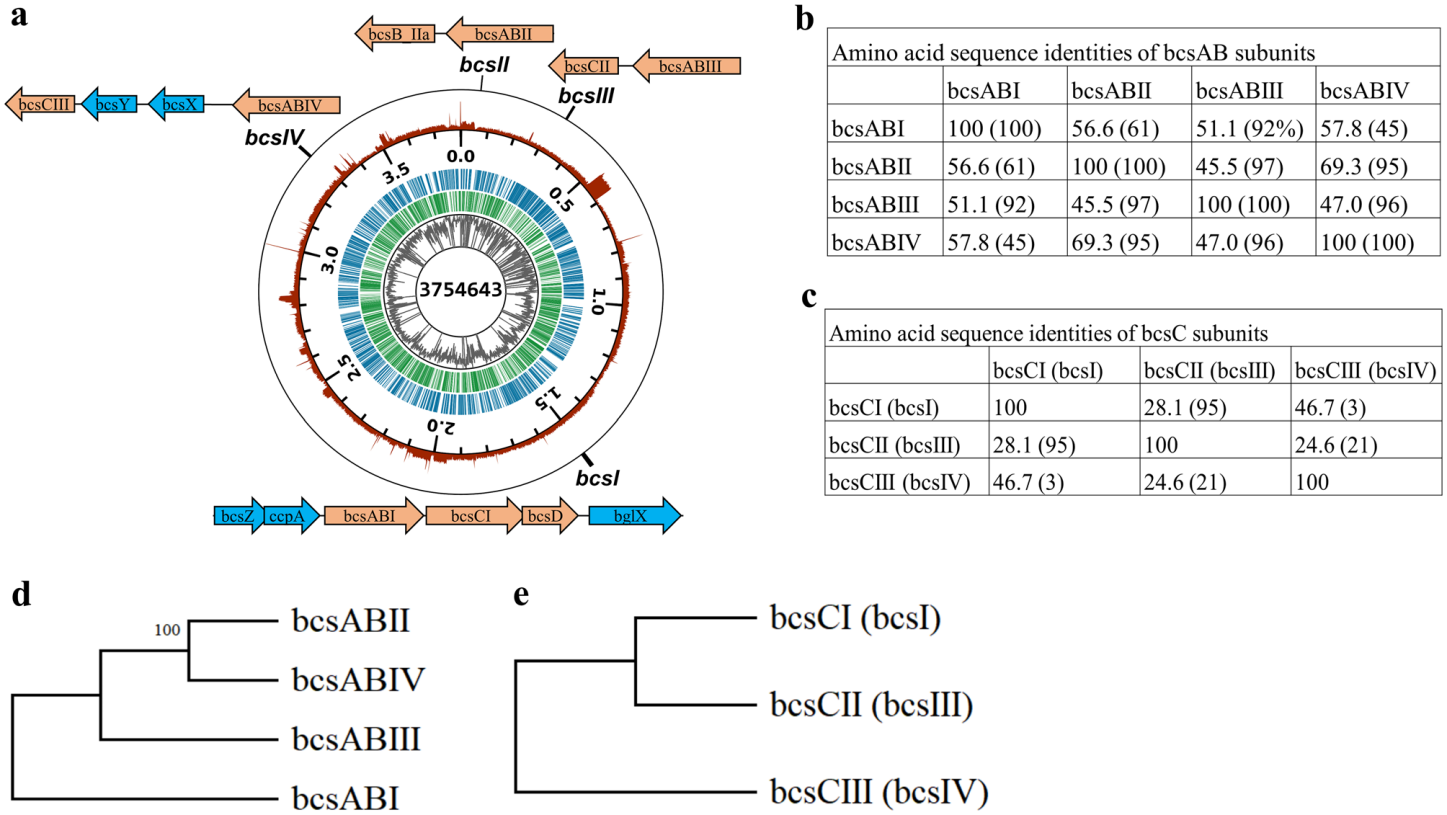
*K. intermedius* ENS15 genome. (**a**) Genome map. The genome contains one chromosome of 3,754,643 bp (GC content 61.8%) with a predicted total of 3467 genes, 120 repeat DNA, 47 tRNAs and 19 non-coding RNA. The displayed data from perimeter to center are, coverage (red), chromosome position (major ticks 500 kbp, minor ticks 100 kbp), forward strand CDSs (in the middle, blue), reverse strand CDSs (in the middle, green), GC-percentage (gray), and chromosome size (bp). The GC-percentage ranged from 17.6 to 69.8% and coverage (number of reads mapping to the locus) from 653 to 14,700. Both GC-percentage and coverage were calculated with 1 kbp window size. Coverage is displayed with logarithmic scaling. The genome map was prepared by matplotlib^34^ (3.3.4; https://matplotlib.org/3.3.4), pysam 0.15.3 (https://github.com/pysam-developers/pysam), and biopython^35^ (1.78; https://biopython.org/docs/1.78) libraries using python language (3.7.11; https://www.python.org/). The bcs operon and the surrounding genes are indicated by manually curated figures directing to its position in the genome. (**b,c)** and (**d,e)** respectively indicates the amino acid sequence identities (MUSCLE program^29^) and the phylogenetic relationship between the predicted bcsAB and bcsC genes present in genome. The phylogenetic tree was build using MEGA X integrated tool^14^ (https://www.megasoftware.net/, version 10.2.4).

The genome data indicated that the *K. intermedius* ENS15 has one chromosome of 3,754,643 bp (GC content, 61.8%) (Fig. 2) comprising of 3467 coding domain regions (CDSs), 120 repeat DNA, 19 non-coding RNA, 46 tRNAs and 1 rRNA (Table S3). Through manual search along the Prokka annotated files, blastP searches, and domain predictions (NCBI CDD, InterproScan), the genes encoding the proteins for bacterial cellulose synthesis, carbohydrate uptake and metabolism and gluconeogenesis were identified from the genome. Amino acid sequences of the aforementioned enzymes are presented in the Supplementary material.

At least one putative plasmid sequences, pKi (~ 80 Kb), containing genes encoding DNA replication, recombinant and repair, relaxases, DNA-binding transcriptional regulator, type IV bacterial secretion system, transposases and toxin-antitoxin systems were identified with plasmidSPAdes. Although DNA replication machineries could not be determined, two additional putative plasmid sequences pKi2 (22 Kb) and pKi3 (13Kbp), mostly containing genes encoding hypothetical proteins and transposases were identified. Recycler indicated that the draft genome did not contain any plasmid sequences. The presence of plasmid DNA within other *Komagataeibacter* spp. genomes have been reported. For instance, an abundance of plasmid DNA, ranging from four to seven have been identified from *K. rhaeticus* ENS9a, *K. xylinus* E25, *K. medellinensis* NBRC 3288 and *K. nataicola* RZS01 genomes, suggests the phenotypic diversity among the bacterial cellulose producers^36^. Blastn alignments of pKi1 and pKi2 sequences with the NCBI non-redundant nucleotide collection of *Komagataeibacter* taxid (id, 1,434,011) indicated similarities to plasmids present in *K. medellinensis* NBRC 3288 (pGXY010, 96% identity and 81% coverage) and *K. xylinus* E25 (pGX5, 98% identity and 88% coverage) strains, respectively.

#### Predicted BC biogenesis machineries

The BC synthetase complex, spanning the outer and inner membranes involved in BC synthesis and export to extracellular milieu, and the adjoining gene clusters present in the *K. intermedius* ENS15 genome is illustrated in Fig. 2a. Four *bcs* operons were annotated in *K. intermedius* ENS15. As previously reported for *Komagataeibacter* spp^4,5,9,10,37^, only one operon contained the complete genes set encoding the BC biogenesis machinery (*bcsAI*, *bcsBI*, *bcsCI* and *bcsD*) was found in *K. intermedius* ENS15 genome (genomic position 1,511,250:1,520,353 bp). Though annotated as individual genes, a manual search identified a 14 bp overlap within *bcsAI* and *bcsBI* genes (*bcsABI*) and 1 bp overlap between *bcsCI* and *bcsD*. The genes encoding the BC biogenesis accessory proteins endoglucanase (BcsZ) and cellulose complementing factor A (CcpA), observed as gene fusions, and beta-glucosidase (BglX) were identified at proximal upstream and downstream regions of *bcsI a*t genomic positions 1,508,964:1,511,059 bp and 1,520,578:1,522,779 bp, respectively. Furthermore, a standalone copy of *bcsZ* gene (*bcsZII*; 28% amino acid sequence identity to BcsZ with a 23% coverage) was identified at position 2,042,498:2,043,523 bp.

Three additional copies of *bcs* operon were present in complement in the genome. The second *bcs* operon (*bcsII*) found at genomic position 60,840:64,661 bp, contained fused *bcsAII* and *bcsBII* genes (*bcsABII*). In silico analysis in NCBI conserved domain database, identified BcsABII to contain domain architectures to glycosyltransferases and BcsB superfamily, confirming the annotation. An interesting observation was the presence of a gene annotated to encode cyclic di-GMP binding protein 31 bps upstream of *bcsABII* (genomic position 60,041:60,808; Fig. S4), which has not yet reported in *Komagataeibacter* spp. BlastP search of the gene against *Komagataeibacter* taxid (id:1434011) resulted in close alignments with *K. intermedius* AF2 cation tolerance protein (100% similarity; accession number KPH88887.1) and *K. oboediens* cellulose synthase catalytic subunit (96% similarity, Accession Number GCE79538.1). NCBI CDD and InterProScan searches predicted the amino acid sequence to contain domain hits for BcsB super family and cellulose synthase, subunit B (IPR003920), allowing to annotate the predicted gene as *bcsB_IIa*. Nevertheless, further validations are required as the annotation could be attributed to the artefacts in the prediction tools which maintains the old annotation of BcsB with regards to c-di-GMP binding. A translated blast search of BcsII_a against the nucleotide sequences of homologous *bcsB* gene from *Komagataeibacter* spp. demonstrating 39–41% sequence similarity (89% query coverage; Fig. S5), supports the hypothesis. Alternatively, such annotations could be related to incorrectly predicted CDS caused by a mutation within the downstream *bcsAB* reading frame. This is crucial to validate as such mutations can lead to premature termination at the transcript-level.

The third *bcs* operon (*bcsIII*) contained fused *bcsAB* genes (*bcsABIII*) at genomic position 335,426:339,913 bp. A second copy of *bcsC* gene (*bcsCII*) was found upstream of the *bcsABIII* gene (position 331,628:335,332 bp). The fourth bcs operon (*bcsIV*) comprising of fused *bcsAB* (*bcsABIV*), genes encoding for SGNH/GDSL hydrolase family protein (BcsX), acyltransferase (BcsY) and BcsC (*bcsCIII*) was found at genomic position 3,293,852:3,304,158 bp. Though the exact function of BcsX and BcsY proteins have not been yet characterized, presence of genes encoding for the stated proteins have been reported in *Komagataeibacter* spp^9,38^.

Variable copy numbers of *bcs* operon genes have been reported among *Komagataeibacter* spp. For instance, four copies of *bcs* operon genes have been identified in *K. intermedius* AF2 (draft genome, GenBank: GCA_000817255.2)^36^, *K. rhaeticus* (NZ_CP050139.1, NZ_LT575493.1)^5,9^ and *K. xylinus* (NZ_CP024644.1) strains, whereas *K. hansenii*, a high yield bacterial cellulose producer, contains three *bcs* operons^4^. In general, apart from the ancillary *bcsB_IIa* gene found at *bcsII*, *K. intermedius* ENS15 shares similar arrangement and composition of *bcs* operon genes as reported for *Komagataeibacter* spp. isolates in the literature. Nevertheless, absence of a complete operon, i.e., *bcsD, bcsZ, ccpA* and *bglX* in *bcsII*–*bcsIV*, suggests that the three additional operon copies might have risen during evolution from duplications and deletion events of *bcsI* operon. Phylogenetic and amino acid sequence identities of BcsAB and BcsC proteins present in the four *bcs* operons are presented in Fig. 2b–e. Phylogenetic analysis of BcsAB proteins suggest *bcsII* and *bcsIV* to be closely related in comparison to the full length *bcsI* (similarity, 56–59%; coverage, 45–61%) and *bcsIII* (similarity, 45–47%; coverage, 96–97%). Whereas *bcsI* and *bcsIII* demonstrated a 51.1% sequence similarity from a coverage of 92%. Similar observation was made from BcsC phylogeny, wherein the highest similarity of 28.1% (coverage 95%) was found among *bcsI* and *bcsIII* operons.

### Insights on carbohydrate metabolism and BC production by *K. intermedius* ENS15

Glucose is an excellent and widely used carbon source for BC production in *Komagataeibacter* spp. Prokka annotations, NCBI CDD domain predictions and blastP searches against the NCBI non-redundant database identified an operon containing the genes encoding phosphoenolpyruvate phosphotransferase (PtsI) system (genomic positions 3,146,276:3,150,394) involved in glucose uptake and utilization^36^. The operon contained, in the order, the genes encoding phosphoenolpyruvate-protein phosphotransferase and the phosphocarrier protein, PTS fructose transporter subunit IIA, and a bifunctional kinase/phosphorylase (involved in catabolite repression and phosphorylation of phosphocarrier protein). Similar to other *Komagataeibacter* spp., *K. intermedius* ENS15 genome did not indicate an annotation for the gene encoding phosphofructokinase (catalysing the conversion of fructose 6 phosphate to fructose 1,6-biphosphate) resulting in an incomplete Embden-Meyerhof-Parnas (EMP or glycolytic) pathway^9,10^. In *Komagataeibacter* spp., glucose oxidation takes two routes, either via glucose-6-phosphate (entering into pentose phosphate pathway, PPP) or through gluconate generation (through the catalytic activity of glucose dehydrogenase). The generated gluconate is either phosphorylated to 6-phophogluconate (entering into central metabolic pathways for biomass formation) or oxidized to ketogluconate (exported to the cultivation medium). Corroborating to the genome information, after a 10-day cultivation period, the *K. intermedius* ENS15 completely utilized glucose generating gluconic acid as the major liquid end-metabolite (6.2 ± 1.8 g/L gluconic acid, corresponding to 31% of the initial glucose concentration), resulting in a final medium pH of 3.2. In addition to the pH drop, gluconate synthesis reduces the glucose availability for BC biosynthesis, as the enzymes phosphoglucomutase and UDP-glucose pyrophosphorylase provides the precursor UDP-glucose moieties to the BC machinery. After the cultivation period, *K. intermedius* ENS15 strain synthesized a BC titer of 1.1 ± 0.1 g/L (yield, 0.7 mg/g_substrate_; BC characterization, Fig. S6 and Table S5). The BC titer obtained from *K. intermedius* ENS15 are comparable to that reported from *K. intermedius* isolates grown in glucose. For instance, Fernández´ et al. (2019) reported a titer of 1.2 g/L BC using *K. intermedius* JF2 isolate and observed a marginal improvement (1.6 g/L) when cultivated in HS medium containing glucose (10 g/L) and mannitol (20 g/L)^39^. In another study, using a base-resistant *K. intermedius* FST213-1 strain, isolated from fermented fruit juice, a BC titer of 2.3 g/L was reported after a 9-day static cultivation in HS medium containing 20 g/L glucose^40^. Table 1 compares the BC titers obtained from this study to that reported for *Komagataeibacter* spp. grown in HS medium containing glucose.

**Table 1:** BC production metrics from *Komagataeibacter* spp. grown statically in growth medium containing glucose, pure glycerol, crude glycerol, and xylose reported in literature.

Bacterial strains	Medium supplements^a^	Production titer (g/L)	References
**Glucose**			
*Komagataeibacter* sp. W1	–	1.2	^41^
*K. rhaeticus* AF1	2% Ethanol	4.1	^3^
*K. rhaeticus* PG2	–	~ 4	^42^
*K. rhaeticus* ENS9a	–	1.4	^9^
*K. rhaeticus* ENS9b	–	0.6	^10^
*G. xylinus*	–	1.3	^43^
*K. xylinus* B-12068	3% Eth	2.2	^44^
*G. xylinus* CGMCC 2955	–	~ 3.3	^45^
*K. hansenii* JR-02	2% Eth	~ 1.2	^46^
*K. hansenii* B22	–	1.6	^7^
*K. intermedius* FST213-1	–	1.2	^40^
*K. intermedius* JF2	–	1.2	^39^
*K. intermedius* ENS15	–	1.1 ± 0.1	This study
**Pure glycerol**			
*Komagataeibacter* sp. W1	–	1.2	^41^
*K. rhaeticus* PG2	–	6.9	^42^
*K. rhaeticus* ENS9a	–	2.6	^9^
*K. rhaeticus* ENS9b	–	3.0	^10^
*Komagataeibacter* sp. nov. CGMCC 17276	30 g/L CSL, 0.3% AA, 1.5% Eth	4.5	^38^
*K. xylinus* B-12068	3% Eth	23.2	
*K. hansenii* JR-02	–	~ 2.4	^46^
*K. intermedius* ENS15	–	1.3 ± 0.0	This study
**Crude glycerol**			
*G. sacchari*	–	0.1	^47^
*Komagataeibacter* sp. nov. CGMCC 17276^b^	30 g/L CSL, 0.3% AA, 1.5% Eth	6.0	^38^
*A. xylinum*	–	~ 1.5	^48^
*G. xylinus* KCCM 41431^c^	–	7.0	^49^
*G. xylinus*	–	~ 1.5	^50^
*K. intermedius* ENS15	–	0.8 ± 0.1	This study
**Xylose**			
*A. hansenii* ATCC 10821	–	40 mg/L	^51^
*A. xylinus* IFO 15606	–	100 mg/L	^51^
*A. pasteurianus* IFO 14814	–	70 mg/L	^51^
*K. intermedius* ENS15	–	400 ± 0.0 mg/L	This study

*CSL* corn steep liquor, *AA* acetic acid, *Eth* ethanol.

^a^Additives to HS medium (5 g/L yeast extract, 5 g/L peptone) reported in respective literature, ^b^BC production using optimized cultivation conditions, ^c^HS medium concentration 9 g/L YE, 9 g/L Pep.

As opposed to glucose, glycerol has been regarded as an ideal substrate for BC biogenesis due to the substrate’s capacity to bypass gluconate generation and improve process sustainability^9,10^. While an annotation specific for glycerol uptake facilitator protein was not present, three gene copies encoding aquaporin Z was identified from the genome. NCBI CDD searches confirmed that among the three copies, two genes encoded aquaporin Z (Genomic Positions 611,611:612,540 bp and 3,112,617:3,113,501 bp) and one showed domain architectures similar to glycerol uptake facilitator (Genomic Position 2,538,382:259,365 bp). Manual search along the predicted *glpF* locations identified genes encoding glycerol kinase (position 2,541,272:2,542,771 bp) and Glycerol-3-phosphate regulon repressor (position 2,544,983:2,545,741), and glycerol dehydrogenase (position 2,412,749:2,414,956 bp) downstream and upstream to *glpF*, respectively. Putative gene encoding dihydroxyacetone kinase was present at genomic location 612,665:614,299 bp, downstream of aquaporin Z_I gene. Furthermore, multiple copies of genes encoding oxaloacetate-decarboxylating malate dehydrogenase (catalysing oxaloacetate to pyruvate reaction) and pyruvate phosphate dikinase (catalysing the transformation of pyruvate to phosphoenol pyruvate) were identified from the genome, hinting possible gluconeogenetic function from the central metabolic pathways^9^. Although literature indicates several reports on BC production from glycerol sources using *Komagataeibacter* spp. (Table 1), to date BC production from unrefined crude glycerol have not been reported using *K. intermedius* strains. In comparison to glucose cultivations, *K. intermedius* ENS15 completely utilized the carbon source, synthesizing 1.3 ± 0.0 g/L BC (yield, 0.7 mg/g_substrate_; BC characterization, Fig. S6 and Table S5). Subsequently, the strain’s ability to utilize crude glycerol, a by-product from biodiesel production process, and synthesize BC was investigated. Replacing pure glycerol with crude glycerol as the carbon source resulted in a 31% drop in BC production titer (Table 1). Crude glycerol, an unrefined glycerol source, obtained from biodiesel production plants often contains impurities such as soaps, fatty acids, ash and other organic compounds, negatively influencing bacterial metabolic processes^9,52^. Thus, the drop in BC production from *K. intermedius* ENS15 could be attributed towards the impurities present in the supplemented substrate. Alternatively, such could be a strain related feature. Although Kose et al. (2013) have reported a BC titer of 3.4 g/L using *Gluconacetobacter intermedius* NEDO-01 strain, in the study a pre-treated crude glycerol fraction was employed for BC biogenesis to eliminate the effects caused by the inhibitory compounds^53^. The study by Carreira et al. (2011) reports crude glycerol toxicity on *G. sacchari*, wherein the toxicity effects were elevated by using a 25–50 fold diluted substrate resulting in a BC titer of 0.1 g/L^47^.

Attempts in employing lignocellulosic biomass as the feedstock for BC production has been hindered by inefficient xylose utilization capabilities of *Komagataeibacter* spp. Early studies on BC biogenesis from xylose was reported by Ishihara et al. (2002), wherein minimal BC titers (ranging from 0 to 100 mg/L) were reported from *K. xylinus* and *K. hansenii* strains (Table 1)^51^. The authors reported that the restrictions were slightly elevated in culture medium containing xylose/xylulose mix resulting in a BC titer of 3 g/L. We identified that *K. intermedius* ENS15 grown in HS medium containing 20 g/L xylose synthesized BC, by far the highest, titer of 400 mg/L. The yeast extract and tryptone present in the HS medium have been reported to influence the BC production among *Komagataeibacter* spp^9,10^. Nevertheless, the low background titers (0.01 ± 0.0 g/L) obtained from HS medium devoid of xylose, encouraged to identify the genes involved in xylose transport and metabolism in *K. intermedius* ENS15. The genome contained genes encoding proteins involved in xylose transport (xylose-proton symporter, genomic position 2,339,051:2,340,538 bp and xylose transporter, genomic position 2,049,024:2,050,391 bp), xylulose kinase (catalyzing the conversion of xylulose to xylulose 5-phosphate, an intermediate in pentose phosphate pathway, genomic positions 2,051,664:2,053,133 bp and 2,673,868:2,675,346 bp) and ribulose-phosphate 3-epimerase. Although blastP searches with amino acid annotations against *E. coli* xylose isomerase did not provide any significant hits, manual searches around the genes encoding xylose transport and xylulose metabolism proteins were conducted. At 9 Kbp upstream of xylulose kinase (2,673,868:2,675,346 bp) a putative gene, annotated by Prokka as a hypothetical protein, was identified to contain domain architectures affiliated to sugar phosphate isomerase/epimerase and xylose isomerase-like superfamily. However, as these domain architectures are shared among several proteins, in depth experimental investigations are required to confirm the hypothesis.

### Plasmid transformation in *K. intermedius* ENS15

**Figure 3:**
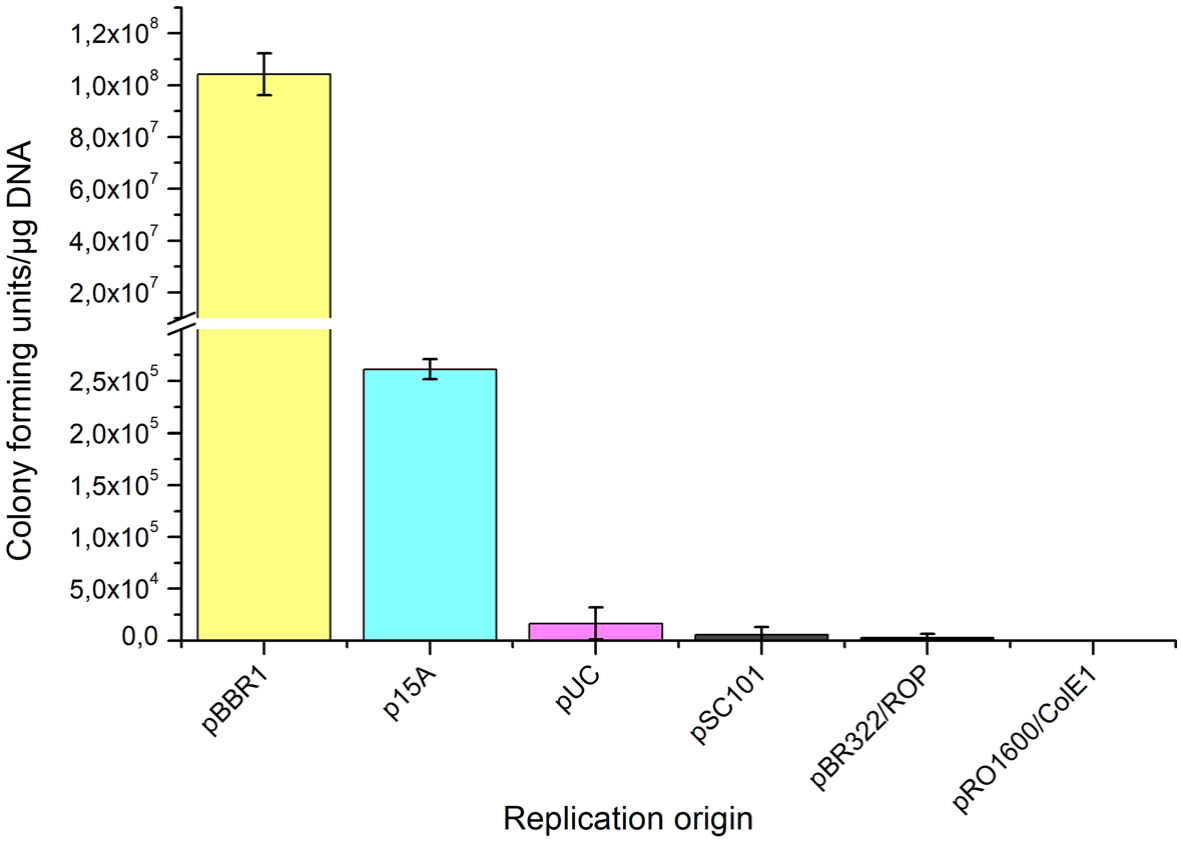
Analysis of variable Ori in *K. intermedius* ENS15. The replication origins pBBR1, p15A, pUC, pSC101, pBR322/ROP and pRO1600/ColE1 are present in pSEVA331, pSEVA361, pSEVA381, pSEVA371, pSEVA391 and pSEVA341, respectively. The presented data includes the mean values and standard deviations from duplicate cultivations in HS-glucose agar containing 340 µg/ml cm.

Plasmid transformation and characterization of genetic engineering toolsets have been studied mostly in *K. xylinus*, *K. hansenii* and *K. rhaeticus* strains^5,6^. Among the plasmids tested, pSEVA321 (RK2 Ori), pSEVA331 (pBBR1 Ori), pSEVA351 (RFS1010 Ori), pBla-Vhb-122 (pBBR1 Ori) and pBAV1C (RepA, modified pWV01 Ori) have been reported to replicate in *Komagataeibacter* spp^4,12^. Here, the genetic tractability of *K. intermedius* ENS15 was studied using SEVA plasmids. Based on the previous studies, pSEVA331 (containing pBBR1 Ori and CmR) was selected as the positive control. SEVA plasmids, pSEVA 211, pSEVA 241, pSEVA 261, pSEVA 271, pSEVA 281 and pSEVA 291, containing KanR and replication origins that have not yet tested in *Komagataeibacter* spp. were chosen in this study (Table S1). In presence of chloramphenicol, wild type *K. intermedius* ENS15 cells was able to grow on HS-glucose agar plates containing 200 µg/ml of the antibiotic and was completely inhibited at 300 µg/ml (Fig. S7A). Since growth inhibition was not observed even at high kanamycin concentrations (700 µg/ml; Fig. S7B), the KanR gene was replaced with CmR from pSEVA331. We identified that *K. intermedius* ENS15 can be transformed via electroporation. Among the tested SEVA plasmids, pSEVA331 (pBBR1 Ori) and pSEVA361 (p15A Ori) showed replication in *K. intermedius* ENS15 (Fig. 3).

### Characterization of constitutive and inducible promoters

**Figure 4:**
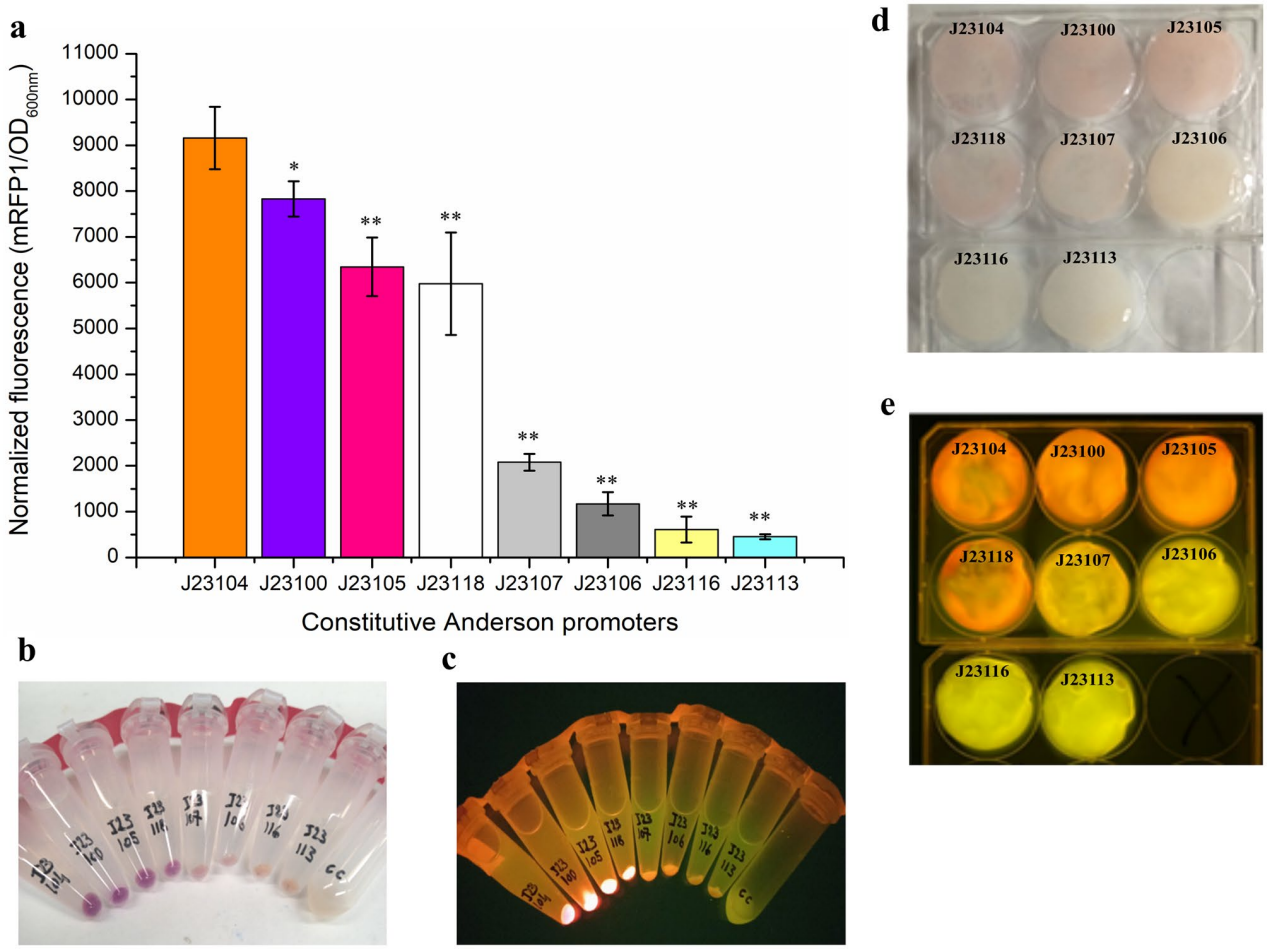
Characterization of constitutive Anderson promoters in *K. intermedius *ENS15. (**a**) strengths of Anderson promoters as measured by normalized fluorescence (mRFP1 fluorescence/OD_600nm_). The promoter strengths were quantified by culturing the recombinant *K. intermedius *ENS15 strains in HS-glucose medium containing chloramphenicol and cellulase for 3 days at 30 °C. The cells were pelleted by centrifugation, washed and resuspended in 1X PBS. The data points represent the mean experimental results and standard deviations from triplicate experimental and technical repeats (n = 6). Statistical significance of the drop in the fluorescence intensity in relation to the data from J23104 was analysed using Two sample t-test in Minitab 19. p < 0.05 and p > 0.05 are represented by * and **, respectively. (**b,c**) Fluorescence from recombinant *K. intermedius *ENS15 cells under normal and blue light, respectively. A control cell (CC) which is the wildtype *K. intermedius *ENS15 is placed for visual comparison in subfigures (**b,c)**. (**d,e**) BC sheets produced by recombinant *K. intermedius *ENS15 strain under normal and blue light, respectively. The images were cropped, and brightness and contrast were adjusted to improve clarity.

Constitutive synthetic *E. coli* promoters from Anderson collection have been characterized in *K. rhaeticus* iGEM, *K. hansenii* ATCC 53582 and *K. xylinus* ATCC 700178^5,6^. Constitutive promoters, J23100, J23106, J23111, J23113, J23116, J23107 and J23118, that have not yet characterized in *Komagataeibacter* spp. were selected in this study. mRFP1 was chosen as reporter to quantify the promoter strength. Based on the data reported by Teh et al.^6^ and Florea et al.^4,5^ J23104 and J23105 promoters reported to confer strong and weak strengths in *Komagataeibacter* spp., were included as positive and negative controls, respectively. For successful propagation in *K. intermedius* ENS15, regulatory elements (promoters, ribosome binding site, mRFP1 and the double terminator) were cloned into pSEVA331 vector. Similar to the reports from *K. rhaeticus* iGEM and *K. hansenii* ATCC 53582, J23104 promoter was identified as the strongest constitutive promoter in *K. intermedius* ENS15 (Fig. 4a). Surprisingly J23105, a weak promoter reported in *K. rhaeticus* iGEM, *K. hansenii* ATCC 53582 and *K. xylinus* ATCC 700178 strains, was found to confer strong fluorescence signal in *K. intermedius* ENS15. The studied promoters showed a similar trend in the mRFP1 signal in both *Escherichia coli* XL1 and *K. intermedius* ENS15 strains with J23104, J23100, J23105, J23118 promoters as the strong promoters (Fig. 4 and Fig. S8). However, a 2-sample t test with J23104 indicated that, except for J23100, the remaining promoters demonstrated a statistically significant drop in the fluorescence signal.

**Figure 5:**
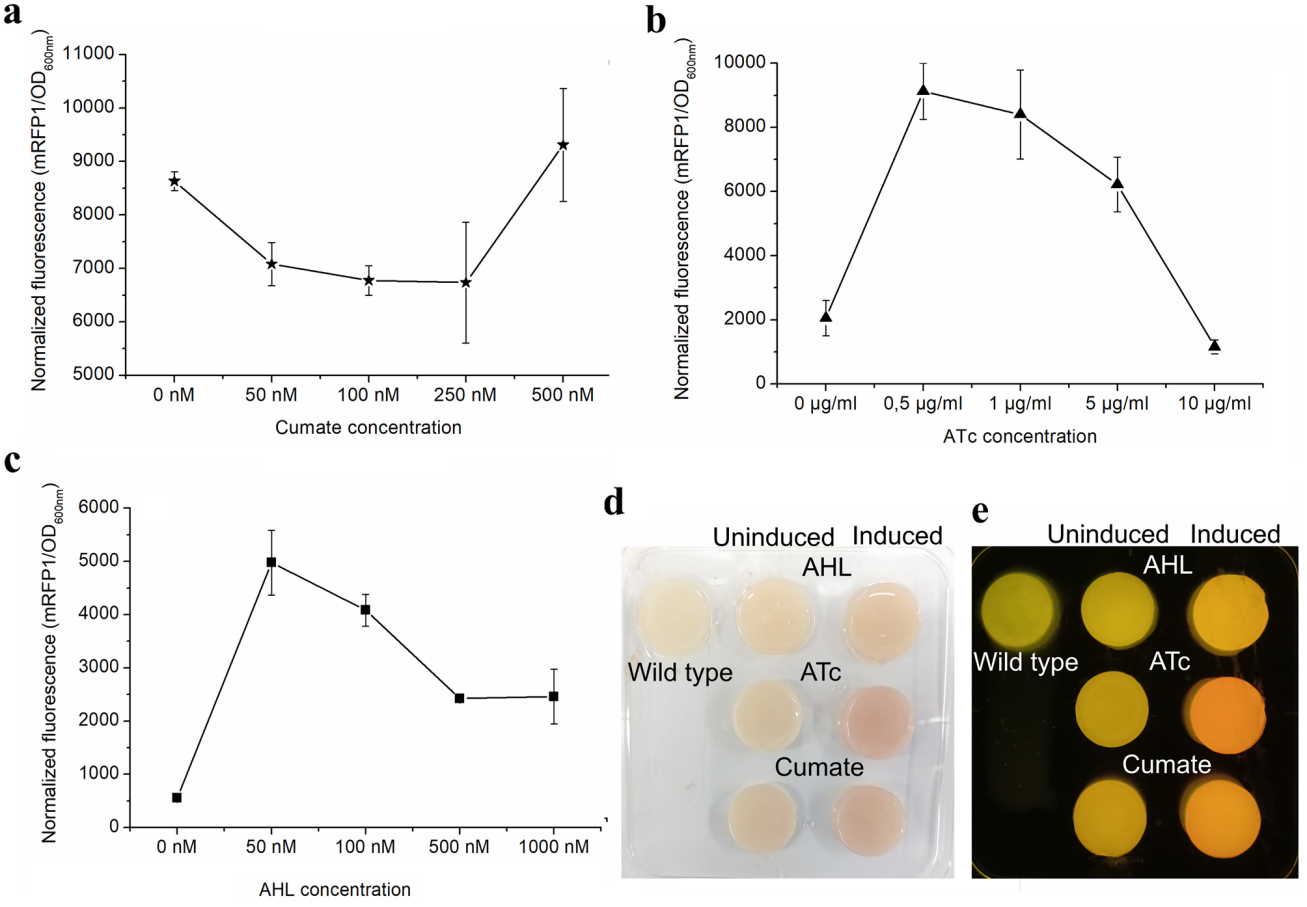
Characterization of inducible constructs in *K. intermedius *ENS15. (**a–c**) mRFP1 expression using AHL-, ATc- and Cumate-inducible constructs as measured by normalized fluorescence (mRFP1 fluorescence/OD_600nm_), respectively. The promoter strengths were quantified by culturing the recombinant *K. intermedius *ENS15 strains in HS-glucose medium containing chloramphenicol, cellulase and varying inducer concentrations for 3 days at 30 °C. The cells were pelleted by centrifugation, washed and resuspended in 1X PBS. The data points represent the mean experimental results and standard deviations from triplicate experimental and technical repeats (n = 6). (**d,e**) BC sheets produced by recombinant *K. intermedius *ENS15 strain under normal and blue light, respectively. For induction within the BC pellicle, single colonies of wild type and recombinant *K. intermedius *ENS15 cells harbouring AHL-, ATc- and Cumate-inducible constructs were precultivated in HS-glucose medium containing 32 µg/mL CmR. The precultivated cells were inoculated, in duplicates, in to 6-well culture plates containing 10 ml of HS-glucose medium containing 32 mg/L CmR and optimal inducer concentration (AHL, 50 nM; ATc, 500 ng/mL; Cumate, 500 nM) and statically grown at 30 °C for 10 days. The images were cropped, and brightness and contrast were adjusted to improve clarity.

AHL-, ATc-, IPTG- and arabinose-inducible systems, under the control of pLux, pTet, pLac and pBAD promoters, have been previously characterized in *Komagataeibacter spp*^5,6^. In the studies, the pLux promoters demonstrated highest mRFP1 expression and less leakiness than the tested counterparts. Due to the lack of *lac* repressors in *Komagataeibacter* genomes, pLac promoter was employed as a constitutive promoter by Teh et al.^6^. Furthermore, in the study, constructs carrying arabinose-inducible pBAD promoter was studied in *Komagataeibacter* spp. In comparison to pLux promoter, the authors identified that the arabinose-inducible construct imparted low mRFP1 expression and identified that the presence of arabinose in the growth medium negatively affected nanocellulose production. Contemplating the literature reports, we investigated mRFP1 expression in *K. intermedius* ENS15 using previously characterized AHL (pLux)- and ATc (pTet)-inducible systems, and a Cumate-inducible expression system that has never been characterized in *Komagataeibacter* spp. (Fig. 5). The Cumate switch consisting of a *cymR* repressor gene and *CuO* repressor-binding operator under strong constitutive promoters have been employed for tight expression of recombinant genes in *E. coli* strains and *Bacillus* spp^33,54^. However, we noted that the Cumate-inducible system was quite leaky in *K. intermedius* ENS15 (Fig. 5a) and the inducer negatively impacted the cell growth (Table S6). Such effect on the cell growth was not observed in *K. intermedius* ENS15 cells harboring AHL- and ATc-inducible constructs, except in the highest ATc concentration (10 µg/ml) which could be attributed towards the tetracycline toxicity (Table S6). In the case of mRFP1 induction using AHL- and ATc-inducible systems in *K. intermedius* ENS15, contradictory results to that published for *K. rhaeticus* iGEM strain was observed. Although AHL-inducible construct imparted low leakiness, a higher induction was observed using ATc-inducible construct with an optimal inducer concentration of 0.5 µg/ml (Fig. 5b). Furthermore, robust induction of mRFP1 expression while encased in the pellicle indicates that, despite entrapped within nanocellulose, the *K. intermedius* ENS15 cells can effectively receive signals from their environment enabling its applicability in applications requiring long-term cell survival and tolerance to toxic compounds (Fig. 5d,e).

## Conclusions

A novel nanocellulose producing bacterium, affiliated to *K. intermedius* LMG 18909^ T^, was isolated from Kombucha SCOBY. The *bcs* operons and accessory genes involved in BC biogenesis and predicted putative genes involved in carbohydrate metabolism was supported by the experimental observation that the isolate metabolized glucose, pure and crude glycerol for biomass formation and BC production. Although BC synthesized from xylose is the highest titer reported from *Komagataeibacter* spp., modest production metrics attributes to a non-efficient route undertaken by the isolate for pentose conversion to gluconeogenesis pathway. The results on successful propagation of SEVA plasmids carrying varying origin of replications and recombinant mRFP1 expression via constitutive and inducible promoter systems indicate that *K. intermedius* strains are genetically tractable. Coupling the obtained empirical data hypothesizes the possibilities of overexpressed native/heterologous genetic systems for improvements in pentose bioconversion to BC. Future studies will involve metabolic engineering attempts using the optimal expression vectors to engineer *K. intermedius* ENS15 strain for efficient xylose bioconversion for BC production.

## Supplementary Information

Supplementary Information.

## Data Availability

All data generated or analysed during this study are included in this published article and in the accompanying supplementary file. The 16S rRNA gene sequence can be found in the NCBI GenBank database under the accession number MT094082. The genome assembly can be found in the NCBI GenBank under the accession number GCA_021555195.1. The protein sequences mentioned in this study can be accessed through the protein FASTA (.fna) file within the GenBank source database. *K. intermedius* ENS15 strain is available from the corresponding author upon request.
