# Supplementary material for: Characterization, genome analysis and genetic tractability studies of a new nanocellulose producing Komagataeibacter intermedius isolate
Source: Sci Rep. 2022 Nov 28;12:20520. doi: 10.1038/s41598-022-24735-z (PMC9705422; doi:10.1038/s41598-022-24735-z)
Supplement: Supplementary file 1 — Supplementary Information. [file 41598_2022_24735_MOESM1_ESM.docx]

# **Characterization, genome analysis and genetic tractability studies of a new nanocellulose producing *Komagataeibacter intermedius* isolate**

Pietro Cannazza ^1^, Antti J Rissanen ^2^, Essi Sarlin ^2^, Dieval Guizelini ^3^, Carlotta Minardi ^2^, Pauli Losoi ^2^, Francesco Molinari ^1^, Diego Romano ^1^, and Rahul Mangayil ^2,4*^

^1^ Department of Food, Environmental and Nutritional Sciences (DeFENS), University of Milan, Via Celoria 2, 20133 Milan, Italy

^2^ Faculty of Engineering and Natural Sciences, Tampere University, Tampere, Finland

^3^ Graduate Program in Bioinformatics, Sector of Professional and Technological Education, Federal University of Parana (UFPR), Curitiba, PR, Brazil

^4^ Department of Bioproducts and Biosystems, Aalto University, Espoo, Finland

* Correspondence: [rahul.mangayil@tuni.fi](mailto:rahul.mangayil@tuni.fi) or [rahul.mangayil@aalto.fi](mailto:rahul.mangayil@aalto.fi)

**Supplementary figures**


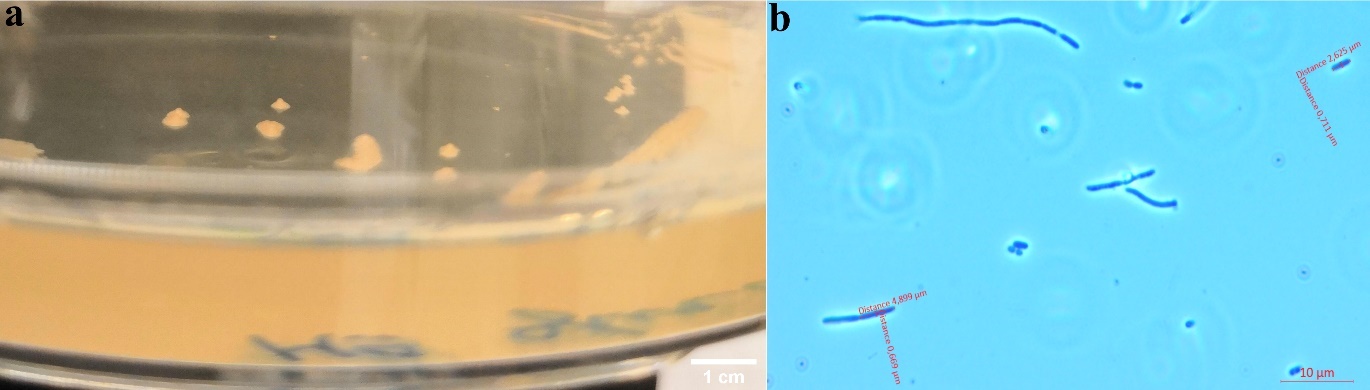


Figure S1. (a) Colony and (b) cell morphologies of ENS15. The images were cropped and brightness and contrast were adjusted to improve clarity





Figure S2. pH changes during cultivations in MA/9 containing 30% glucose and PY medium containing 3% ethanol, 0.35% acetic acid, 3% ethanol and 4% acetic acid. The cultivations were conducted at 30°C and 230 rpm for 14 days. Averaged values and standard deviations from duplicate cultivations are presented. In some cases, the error bars are smaller than the symbol


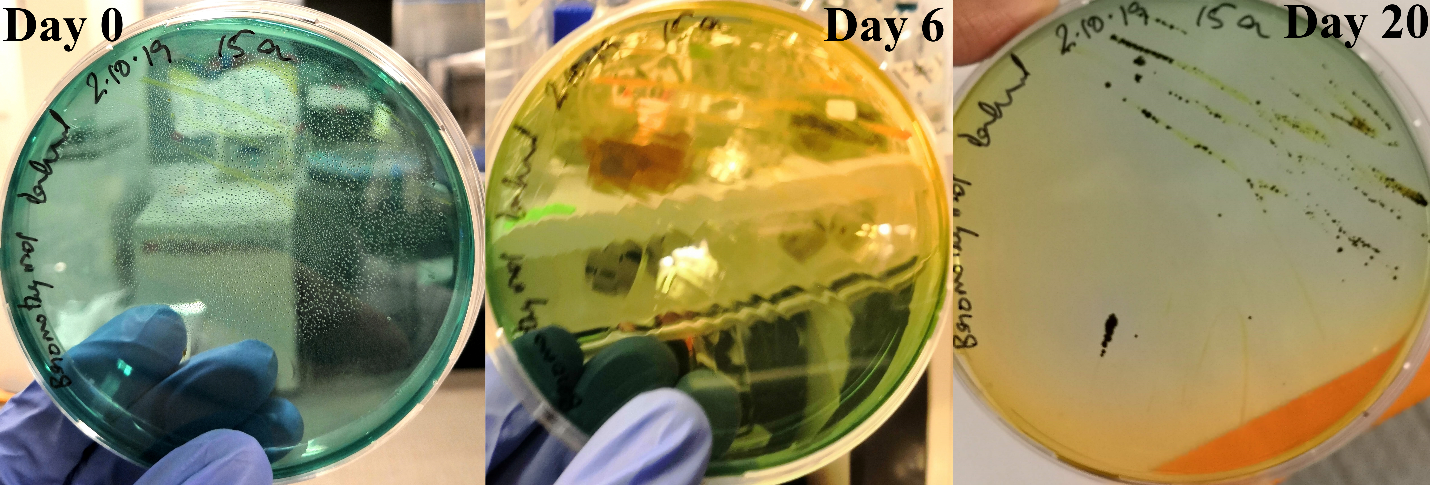


Figure S3. Acetic acid overoxidation. Single colonies were streaked onto PY medium containing 2% ethanol and 0.0002% bromothymol blue as the pH indicator. Figures (from left to right) shows the acidification (blue to yellow coloration) and neutralization (yellow to blue) of PY agar during 0, 6, and 20 days of incubation at 30°C. The images were cropped, and brightness and contrast were adjusted to improve clarity.


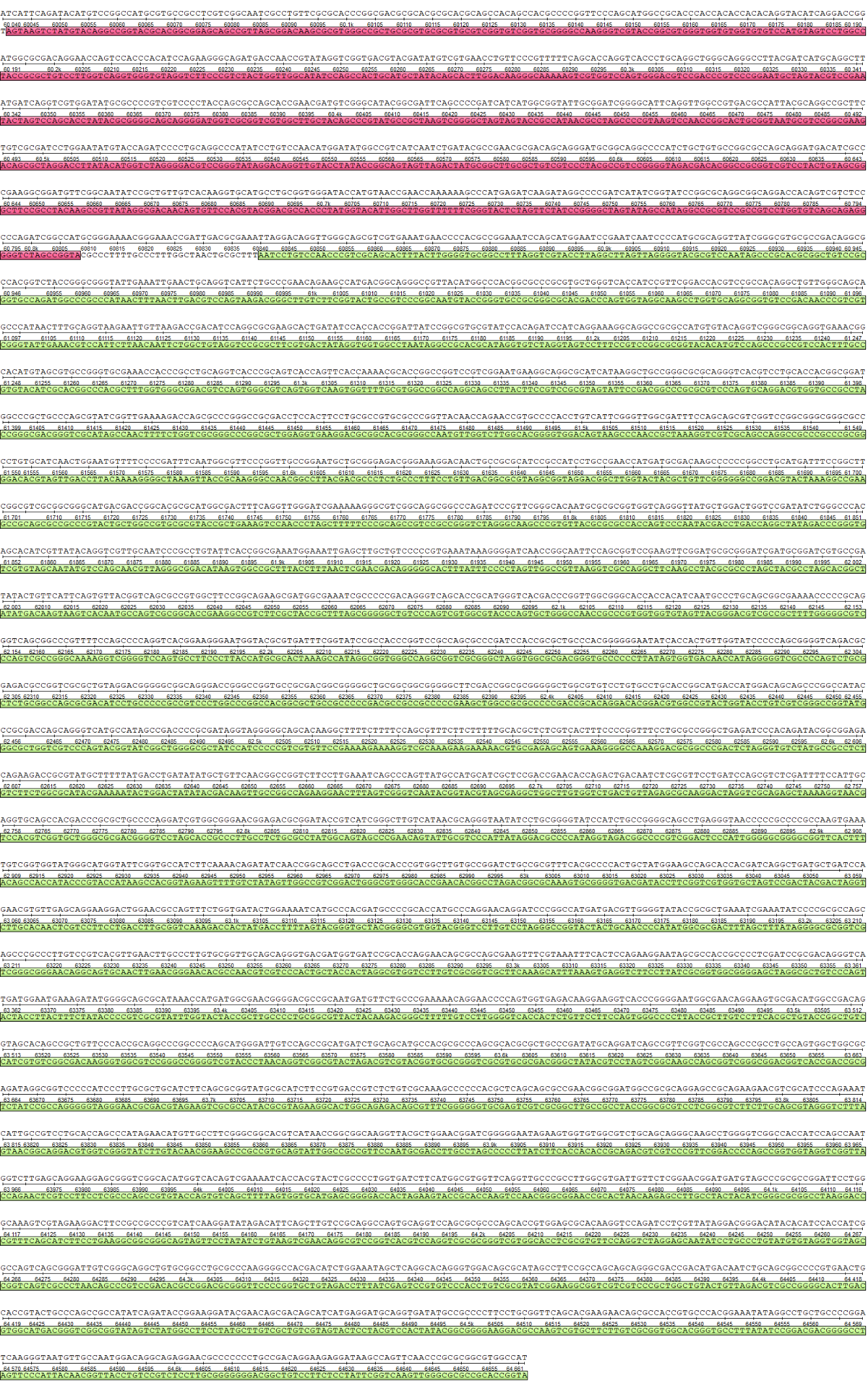


Figure S4. Nucleotide sequence (in complement) within the *K. intermedius* ENS15 genome region covering the predicted *bcsB_IIa* (marked in red) and *bcsABII* (marked in green).


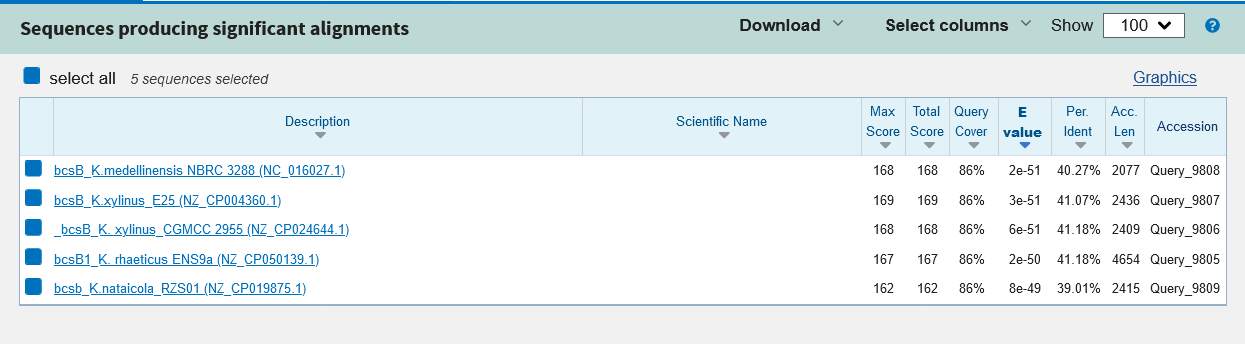
Figure S5. Translated blast search of BcsII_a against the nucleotide sequences of homologous *bcsB* gene from *Komagataeibacter* spp. Genbank accession codes of Komagataeibacter genome are presented in parenthesis.


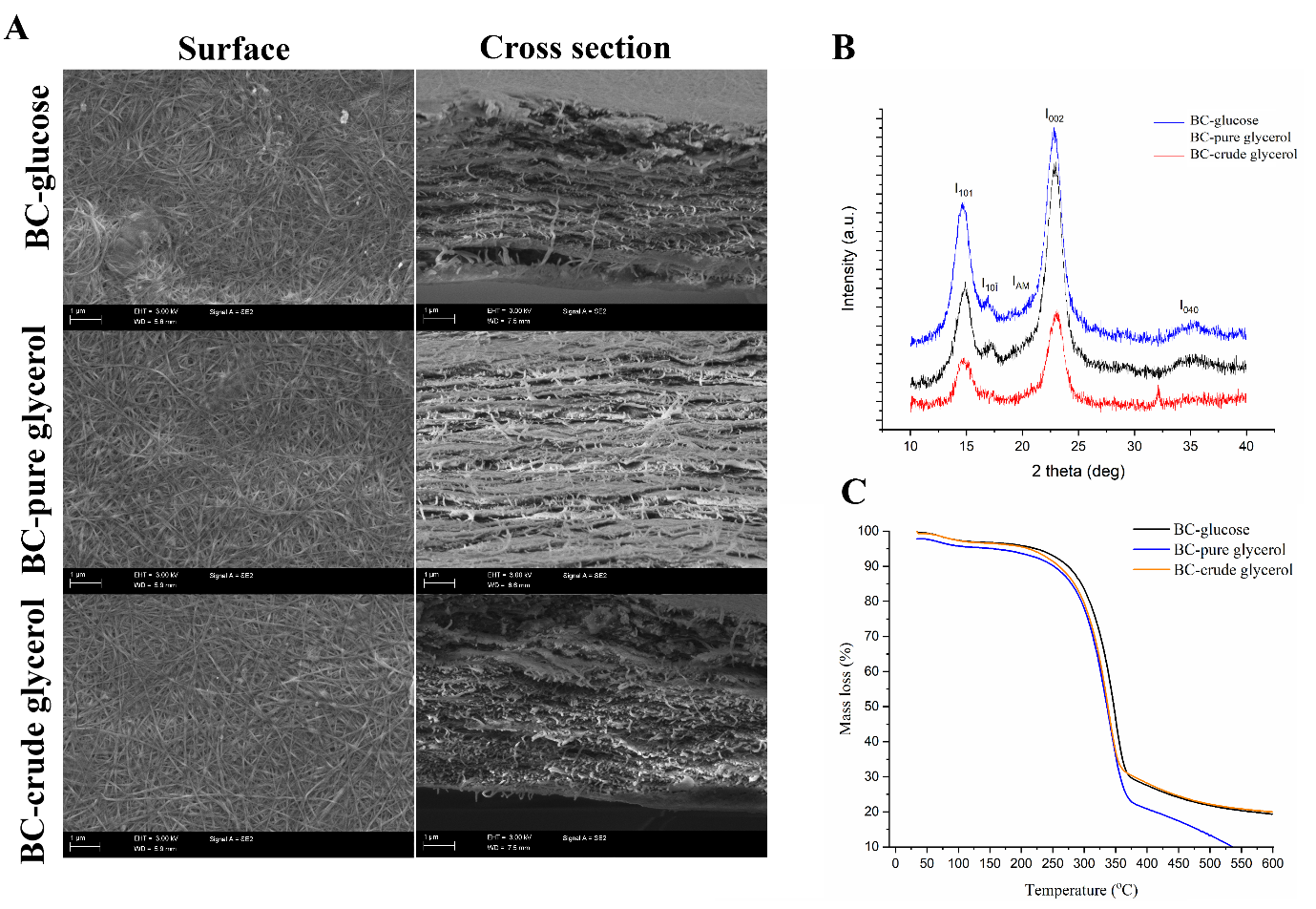


Figure S6. Characterization of BC synthesized from HS-medium supplemented with glucose, pure glycerol and crude glycerol. (A) Surface and cross-sectional images from SEM. (B) XRD diffractograms presenting the diffraction peaks at I_101_ and I_10_ī, I_002_ and I_040_, and I_AM_ indicating the crystalline Iα, Iβ and amorphous regions in BC. (C) Thermal characteristics analysed using TGA. Due to the fragile nature, the BC synthesized from xylose was excluded from analysis.


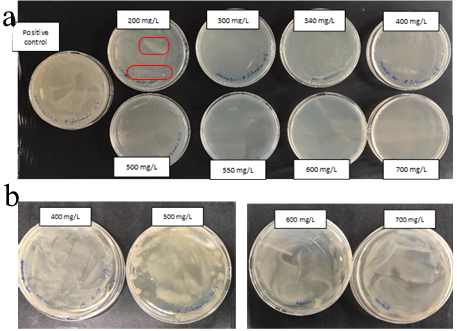


Figure S7. Antibiotic sensitivity tests. *K. intermedius* ENS15 electrocompetant cells streaked on to HS-gluc agar plates containing varying concentrations of (A) chloramphenicol and (B) kanamycin and incubated at 30°C for 5 days. The cell grown in agar plate containing 200mg/L of chloramphenicol is highlighted in red.


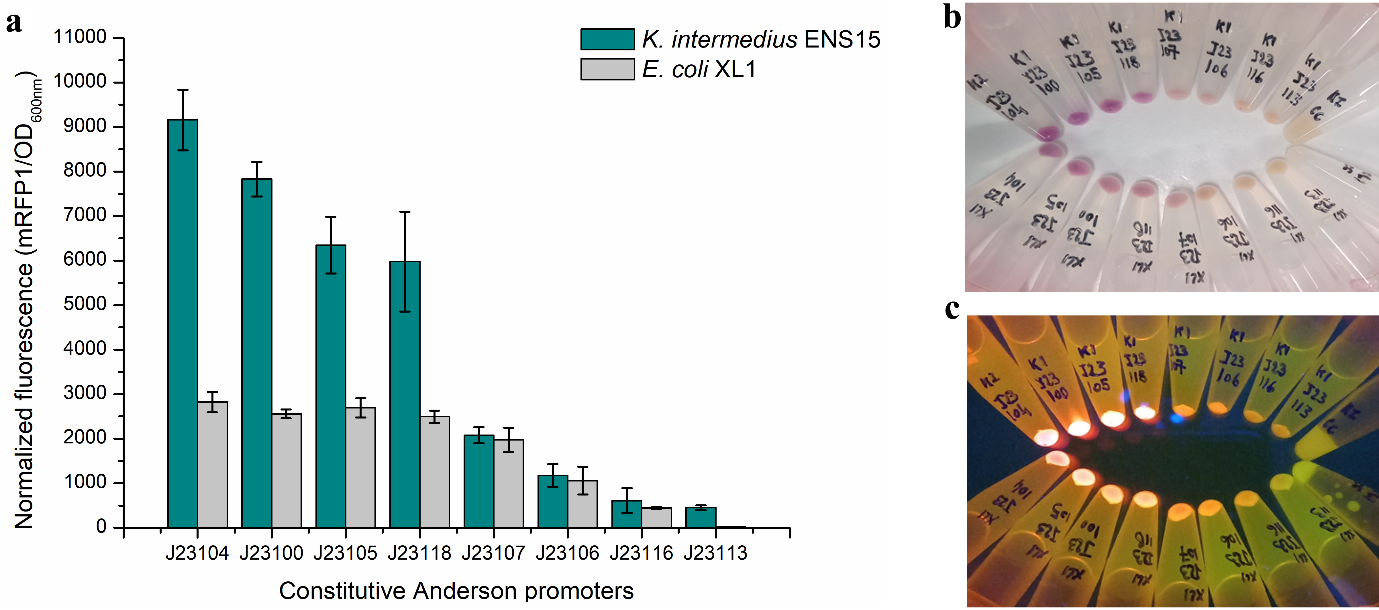


Figure S8. Characterization of constitutive Anderson promoters in *K. intermedius* ENS15 and *E. coli* XL1 as measured by normalized fluorescence (mRFP1 fluorescence/OD600nm). (B and C) Fluorescence from recombinant *K. intermedius* ENS15 (KI, top) and *E. coli* (XL1, bottom) cells under normal and blue light, respectively.

**Supplementary Tables**

| Table S1. Plasmids used in this study | | | | |
| --- | --- | --- | --- | --- |
| Plasmid | Origin of replication | Antibiotic resistance | Feature | Repository |
| pSEVA211 | R6K | Kanamycin | Empty vector | SEVA-DB |
| pSEVA**3**11 | R6K | Chloramphenicol | Empty vector | This study |
|  |  |  |  |  |
| pSEVA241 | pRO1600/ColE1 | Kanamycin | Empty vector | SEVA-DB |
| pSEVA**3**41 | pRO1600/ColE1 | Chloramphenicol | Empty vector | This study |
|  |  |  |  |  |
| pSEVA261 | p15A | Kanamycin | Empty vector | SEVA-DB |
| pSEVA**3**61 | p15A | Chloramphenicol | Empty vector | This study |
|  |  |  |  |  |
| pSEVA271 | pSC101 | Kanamycin | Empty vector | SEVA-DB |
| pSEVA**3**71 | pSC101 | Chloramphenicol | Empty vector | This study |
|  |  |  |  |  |
| pSEVA281 | pUC | Kanamycin | Empty vector | SEVA-DB |
| pSEVA**3**81 | pUC | Chloramphenicol | Empty vector | This study |
|  |  |  |  |  |
| pSEVA291 | pBR322/ROP | Kanamycin | Empty vector | SEVA-DB |
| pSEVA**3**91 | pBR322/ROP | Chloramphenicol | Empty vector | This study |
|  |  |  |  |  |
| pSEVA331 | pBBR1 | Chloramphenicol | Empty vector | SEVA-DB |
| pSEVA331Bb | pBBR1 | Chloramphenicol | Biobrick multiple cloning site | Addgene (#78269) |
| J23104-mRFP1-331Bb | pBBR1 | Chloramphenicol | pSEVABb containing mRFP1 gene downstream of J23104 constitutive promoter |  |
|  |  |  |  |  |
| pSB1C3-J23100 | pMB1 | Chloramphenicol | Constitutive promoter | iGEM Parts Registry |
| pSEVA331- J23100 | pBBR1 | Chloramphenicol | Constitutive promoter | This study |
|  |  |  |  |  |
| pSB1C3-J23105 | pMB1 | Chloramphenicol | Constitutive promoter | iGEM Parts Registry |
| pSEVA331- J23105 | pBBR1 | Chloramphenicol | Constitutive promoter | This study |
|  |  |  |  |  |
| pSB1C3-J23106 | pMB1 | Chloramphenicol | Constitutive promoter | iGEM Parts Registry |
| pSEVA331- J23106 | pBBR1 | Chloramphenicol | Constitutive promoter | This study |
|  |  |  |  |  |
| pSB1C3-J23107 | pMB1 | Chloramphenicol | Constitutive promoter | iGEM Parts Registry |
| pSEVA331- J23107 | pBBR1 | Chloramphenicol | Constitutive promoter | This study |
|  |  |  |  |  |
| pSB1C3-J23113 | pMB1 | Chloramphenicol | Constitutive promoter | iGEM Parts Registry |
| pSEVA331- J23113 | pBBR1 | Chloramphenicol | Constitutive promoter | This study |
|  |  |  |  |  |
| pSB1C3-J23116 | pMB1 | Chloramphenicol | Constitutive promoter | iGEM Parts Registry |
| pSEVA331- J23116 | pBBR1 | Chloramphenicol | Constitutive promoter | This study |
|  |  |  |  |  |
| pSB1C3-J23118 | pMB1 | Chloramphenicol | Constitutive promoter | iGEM Parts Registry |
| pSEVA331- J23118 | pBBR1 | Chloramphenicol | Constitutive promoter | This study |
|  |  |  |  |  |
| pLux mRFP1 | pBBR1 | Chloramphenicol |  | Addgene (#78281) |
| pTet mRFP1 | pBBR1 | Chloramphenicol |  | Addgene (#78283) |
|  |  |  |  |  |
| pCT5-bac2.0 | pBR322 | Ampicillin |  | Addgene (#119872) |

| Table S2. Primers used in this study | |
| --- | --- |
| Primer | Sequence |
| PS5_SEVA | 5’- CCCTGCTTCGGGGTCATT-3’ |
| PS6_SEVA | 5’- GGACAAATCCGCCGCCCT -3’ |
| Fwd_CmR | 5’-CTATCGCTAGCATGGAGAAAAAAATCACTGG-3’ |
| Rev_CmR | 5’- CTAGTCTAGACAAAAGTCAAATTACGCCC-3’ |
|  |  |
| ecoRI_cumate | 5’-gcGAATTCtagttcacactggctcaccttcgggt-3’ |
| xbaI_cumate | 5’- gcTCTAGAcgccggcatgagctcctctagtagag-3’ |

| Table S3. General genome and assembly statistics of K. intermedius ENS15 genome | |
| --- | --- |
| Attribute | Value |
| Total sequence length | 3,869,279 bp |
| Number of contigs | 74 |
| N50 (shortest sequence length at 50% of the genome) | 158,434 bp |
| L50 (smallest number of contigs comprising of 50% of the genome) | 9 |
| Bowtie2 mapping | 93.5% |
| Chromosome | 3,754,643 bp (GC content, 61.8%) |
| Origin of replication^a^ | 3154258:3154664 bp |
| Number of protein-coding genes ^b^ | 3467 |
| Number of repeat DNA ^c^ | 120 |
| Number of rRNA ^b^ | 1 |
| Number of tRNA ^b^ | 47 |
| Number of ncRNA ^b^ | 19 |
| Predicted plasmids | 3; pKi1 (79,597 bp), pKi2 (21,795 bp), pKi3 (13,244 bp) |

^a^ Identified using Ori-Finder tool (http://tubic.org/Ori-Finder/, accessed on 20.06.2021) ^1^.

^b^ Predicted from Prokka using the in-built the feature tools (Prodigal, RNAmmer, Aragorn, Infernal) ^2^.

^c^ Predicted using the Tandem Repeats Finder (https://tandem.bu.edu/trf/trf.html, accessed on 26.06.2021) ^3^.

| Table S4. Pairwise comparisons of *K. intermedius* ENS15 genome against user defined (in bold) and type strain genomes in TYGS server. The digital DNA-DNA hybridization values (d_0_, d_4_ and d_6_) are provided along with their confidence intervals (C.I.) for the three different genome BLAST distance phylogeny formulas. | | | | |
| --- | --- | --- | --- | --- |
| Subject | d_0_ ^a^ (C.I) % | d_4_ ^b^ (C.I) % | d_6_ ^c^ (C.I) % | G+C content difference % |
| ***K. intermedius* TF2** | 86.9 (83.3 - 89.8) | 99.2 (98.8 - 99.5) | 91.6 (89.0 - 93.6) | 1.05 |
| ***K. intermedius* AF2** | 78.6 (74.7 - 82.1) | 97.1 (96.0 - 97.9) | 84.6 (81.4 - 87.4) | 1.29 |
| *K. oboediens* LMG 18849 | 59.5 (55.9 - 63.1) | 61.7 [58.8 - 64.5] | 61.2 [57.9 - 64.3] | 1.28 |
| *K. swingsii* LMG 22125 | 42.5 [39.1 - 45.9] | 41.9 [39.4 - 44.5] | 41.8 [38.8 - 44.8] | 0.23 |
| *K. europaeus* LMG 18890 | 40.2 [36.8 - 43.6] | 42.3 [39.8 - 44.9] | 39.8 [36.8 - 42.8] | 1.39 |
| *K. swingsii* LMG 22125 | 42.5 [39.1 - 45.9] | 41.9 [39.4 - 44.5] | 41.8 [38.8 - 44.8] | 0.23 |
| *K. diospyri* MSKU 9T | 38.4 [35.0 - 41.9] | 39.6 [37.1 - 42.1] | 37.7 [34.8 - 40.8] | 2.22 |
| *K. xylinus* NBRC 15237 | 25.7 [22.3 - 29.3] | 33.9 [31.5 - 36.4] | 25.5 [22.7 - 28.6] | 0.35 |
| *K. medellinensis* NBRC 3288 | 23.3 [20.1 - 27.0] | 33.4 [31.0 - 35.9] | 23.4 [20.5 - 26.5] | 2.07 |
| *K. melomenusus* AV436 | 25.3 [21.9 - 28.9] | 33.2 [30.8 - 35.7] | 25.1 [22.2 - 28.2] | 0.23 |
| *K. sucrofermentans* LMG  18788 | 25.3 [22.0 - 29.0] | 33.2 [30.8 - 35.7] | 25.2 [22.3 - 28.3] | 0.3 |
| *K. nataicola* LMG 1536 | 24.8 [21.5 - 28.4] | 33.2 [30.8 - 35.8] | 24.7 [21.8 - 27.8] | 1.16 |
| *K. melaceti* AV382 T | 25.6 [22.3 - 29.2] | 33.0 [30.6 - 35.5] | 25.4 [22.5 - 28.5] | 0.08 |
| *K. rhaeticus* LMG 22126 | 27.4 [24.1 - 31.0] | 32.3 [29.9 - 34.8] | 26.9 [24.0 - 30.0] | 0.86 |
| *K. saccharivorans* LMG  1582 | 23.7 [20.4 - 27.4] | 32.0 [29.6 - 34.5] | 23.6 [20.8 - 26.7] | 1.03 |
| *K. pomaceti* T5K1 | 18.3 [15.2 - 21.9] | 31.7 [29.3 - 34.2] | 18.6 [15.9 - 21.6] | 0.09 |

^a^ Genome to genome distance calculator formula 1 calculated using the length of all high-scoring segment pair (HSP) divided by total genome length; ^b^ Genome to genome distance calculator formula 2 calculated using the sum of all identities found in HSPs divided by overall HSP length; ^c^ Genome to genome distance calculator formula 3 calculated using the sum of all identities found in HSPs divided by total genome length.

| Table S5. Crystallinity indices (CI) of BC synthesized from HS-medium containing 2% of glucose, pure glycerol and crude glycerol | |
| --- | --- |
| Sample | CI ± standard deviation |
| BC-glucose | 74±5 % |
| BC-pure glycerol | 83±16 % |
| BC-crude glycerol | 76±2 % |

| Table S6. Recombinant *K. intermedius* ENS15 growth (OD_600nm_) at varying inducer concentrations. | | | | |
| --- | --- | --- | --- | --- |
| AHL concentrations | | | | |
| 0 nM | 50 nM | 100 nM | 500 nM | 1000 nM |
| 3.0±0.3 | 2.6±0.1 | 3.3±0.5 | 3.2±0.1 | 3.0±0.2 |
| ATc concentrations | | | | |
| 0 µg/ml | 0.5 µg/ml | 1 µg/ml | 5 µg/ml | 10 µg/ml |
| 3.1±0.4 | 3.5±0.4 | 4.0±0.9 | 3.1±1.2 | 1.3±0.2 |
| Cumate concentrations | | | | |
| 0 nM | 50 nM | 100 nM | 250 nM | 500 nM |
| 2.3±0.0 | 1.1±0.2 | 0.5±0.0 | 0.5±0.0 | 0.5±0.0 |

**Amino acid sequences**

Proteins involved in BC biogenesis

>*bcsABI*

MSEVQSSAPAESWFGRFSNKILSLRGASYVVGALGLCALLAATMVTLSLNEQMIVALVCVAVFFIVGRRKSRRTQVFLEVLSALVSLRYLTWRLTETLDFDTWTQGVLGVTLLLAELYALYMLFLSYFQTISPLHRAPLPLPANPDDWPTVDIFIPTYDEALSIVRLTVLGALGIDWPPDKVNVYILDDGRREEFARFAEACGARYIARPDNAHAKAGNLNYAIKHTTGDHILILDCDHIPTRAFLQIAMGWMVSDSKIALLQTPHHFYSPDPFQRNLAVGYRTPPEGNLFYGVIQDGNDFWDATFFCGSCAILRRKAIEEIGGFATETVTEDAHTALRMQRKGWSTAYLRIPLASGLATERLITHIGQRMRWARGMIQIFRVDNPMLGPGLKLGQRLCYLSAMTSFFFAIPRVIFLASPLAFLFFSQNIIAASPLAVGVYAIPHMFHSIATAAKVNKGWRYSFWSEVYETVMALFLVRVTIVTMLFPSKGKFNVTEKGGVLEREEFDLTATYPNIIFAGIMALGLLRGVYALIFQHLDIISERAYALNCIWSVISLIILMAAISVGRETKQLRQNHRIEAQIPVTVYDYDGNSSHGITEDVSMGGVAIHLPWREVTPDQPVQVVIHAVLDGEEMNLPAIMTRSARGKAVFTWSISNIQVEAAVVRFVFGRADAWLQWNNYEDDRPLRSLWSLILSIKALFRRKGQMIAHSRPKKKPIALPVERREPTTSQGGQKQEGKISRAASMRPRDMKMVSLIALLVFATGAQAAPIASKAPAHQPTGSDLPPLPAAAPVAPAAQPSAQATDPASAAPASDAGSASNADAILDNAENAAGVGTDVATVHTYSLQELGAQSALTMRGAAPLQGLQFGIPADQLVTSARLVVSGAMSPNLQPDNSAVTITLNEQYIGTLRPDPTHPAFGPLSFDINPIFFVSGNRLNFNFASGSKGCADPTNGLQWASVSEHSQLQITTIPLPPRRQLARLPQPFFDKTVRQKVVIPFVLAQTFDPEVLKASGIIASWFGQQTDFRGVNFPVFSTIPQTGNAIVVGVADELPAALGRPSVSGPTLMEVANPSDPNGTVLLVTGRDRDEVIAASKGIGFGSSALPVASRMDVAPIDVAPRQANDAPSFIPTSRPVRLGELVPVSALQGEGYTPGVLSVPFRVSPDLYTWRDRPYKLNVRFRAPDGPILDVARSHLDVGINNTYLQSYSLREQSSVVDQLLHRVGVGTQNAGVEQHTLTIPPWMVFGQDQLQFYFDAAPLAQPGCRPGPSLIHMSVDPDSTIDLSNAYHITRMPNLAYMASAGYPFTTYADLSRSAVVLPDHPNGTVVSAYLDLMGFMGATTWYPVSGVDIVSADHVSDVADRNLIVLSTLSNSADVSALLANSAYQISDGRLHMGLRSTLSGVWNIFQDPMSVMSNTHPTEVETTLSGGVGAMVEAESPLASGRTVLALLSGDGQGLDNLVQILGQRKNQAKVQGDLVLAHGDDLTSYRSSPLYTVGTVPLWLMPDWYMHNHPFRVIVVGLAGCLMVVAVLVRALFRHAMVRRRQLQEERQKS

>*bcsCI*

MNRRYVFSLSAGLLASSCMGAMMPVPVARAQQASTAMTGAQATGGAVAPRQILLQQARFWLQQQQYDNARQALQNAQRIAPDAPDVLEVQGEYQTAMGNREAAADTLRHLQQVAPGSVAANSLSDLLHERSISTGDLSHVRSLAASGHNAEAVAGYQKLFNGGRPPHSLAIEYYQTMAGVPADWDQARAGLSGLVAANPQDYRAQLAFAQTLTYNTSTRMEGLARLKDLQGFRTQAPVEAAAAAQSYRQTLSWLPVTAETQPLMQQWLAAHPDDTALKDHMLHPPGGPPDKAGLARQAGYQQLNAGRLSAAEQSFQSALQINSHDADSLGGMGLVSMRQGDAAEARRYFQEAMAADPKTADRWRPALAGMEISGDYAAVRQLIAAHQYTEAKQRLTSLARQPGQFTGATLMLADLQRSTGQIDASEQEYRSVLARDPNNQLALMGLARVDMAQGNTAEARQLLSRVGPQYATEVGEIEVTGLMAAASHTSDSARKVAILREAMTQAPRDPWVRINLANALQQQGDVAEAGRVMQPILANPVTAQDRQAGILYTYGAGNDAATRRLLSGLSPEDYSPAIRSIAEEMQIKEDLASRLSMVPNPVPLIREALAPPDPTGARGVAVADLFRQRGDMIHARMALRIASTRTIDLSPDQRLAYATEYMKISNPVAAARLLAPLGDGSGSGAGNALLPEQQQTLQQLRMGIAVAQSDLLNQRGDQAQAYDHLAPALRADPEATSPKLALARLYNGEGKSSKALDIDLAVLRHNPQDLDARQAAVQAAVNSGRKSLATHLAMDGVQESPMDARAWLGMAVADQADGHGHRTIADLRRAYDLRLQQVEGARSAFGPAATEEEALAPPSSNPFRHHGYGRQTELGAPVTGGSYSMEATSPEAADQMLSSISGQINTLRENLAPSIDGGLGFRSRSGEHGMGRLTEANIPIVGRLPLQAGESSLTFSITPTMIWSGDLNTGSVYDVPRYGTNMATQAYNQYVNSMNQNNSSSSLRTQQIQGGQGEAGFAPDVQFSNSWVRADVGASPIGFPITNVLGGVEFSPRVGPVTFRVSAERRSITNSVLSYGGLRDPNYNSALGRYALNHYGSQLASQWGQEWGGVVTNHFHGQVEATLGNTILYGGGGYAIQTGKNTRSNNEREAGIGANTLVWHNANMLVRIGVSLTYFGYANNQDFYTYGQGGYFSPQSYYSATVPIRYAGQHKRLDWDVTGSVGYQVFHEHSSPFFPTSSLLQAGAQYIADSYVQNATASDYLSEETVDRAYYPGDSIASLTGGFNARVGYRFTHNLRLDLSGRWQKAGNWTESGAMISVHYLIMDQ

>*bcsD*

MTTFNAKPDFSLFLQALSWEIDDQAGIEVRNDLLREVGRGMAGRLQPPLCNTIHQLQIELNALLGMINWGYVKLELLAEEQAMRIVHEDLPQVGSAGEPSGTWLAPVLEGLYGRWITSQPGAFGDYVVTRDVDAEDLNSVPTQTIILYMRTRSNSN

>*bcsZ*

MLLDFMKLQKHVSGMGRRSFLSVMAATGSIPFLSAAEAGDGTAVSQQWAIFRSKYFHPDGRIVDTGNSGESHSEGQGYGMLFAATAGDQAAFEAIWVWARNNLQHKTDALFSWRYLDGHNPPVADKNNATDGDLLIALGLARAGKLWKRADYIQDAINIYADVLKHMTMKVGPYTVLLPGADGFVTKEAVTLNLSYYVMPSLLQAFELSGESQWQTVIENGLRIIGKAQFGEWKLPPDWLSINRQTGNFSIAKGWPPRFSYDAIRVPLYLYWAHMLSPELLADYTRFWNHFGASALPGWVDLTNGSRSPYNAPPGYLAVASCSGLASAGELPTLDHAPDYYSAALTLLVYIARAEGGGM

>*ccpA*

MSSADKDAGTPAPHRNVDMDNPQDVSRMLTTGYGLSGEGFHYHSFRSIVRDTPVDVPEEADHDDTHAYVEEHYAEPEPYASAPAVAPAPEPEPPAVTPVAMPPIVEEAPPPPPPPPPPPPPAPVVPEVVHVPQPPPQPAPPVMETVAPEPPPPSPPPPETVVSPAPQPRPAATTPDVVQSGGRERRGLPPFVAPATPPRPAPAQSASFTIEAPEPEVVATDEWAPVPKAQQRRGQRPTGPGFFFAKAGDRTQMARLFQPTPVPMPRPVSKPASKVTTMTKFDKNSWNESAGRRPAPTDNSPTLTEVFMTLGGRATDRLIPKPSLREALLRKREEENEQS

>*bcsZII*

MTLPHSSRPGRWLCGCLIATALALPPGGHADAQTYRGVNLAGAAYSSSKLPGRYGYDYLYPKPAEVDYFTAQGMNTFRLSVLWERLQPALNGPLDEKELQRVQQFIAYAQGKGATTVLDIHNYGRYRGQEVGSDAVPDAAFGDLWARLAQALGTNPHVLFGLMNEPQQHSAEAWKNAVQAAIDAIRKTGSHNIILVPGIGWDSALGFAKLNGDALGQLHDPDNRLVYEVHEYFDPDASGTKPACISQDQAVGRLKSFTDWLHAHKAHGFLGEFGVSRQPECVALLQPVLSHLRDNADVWSGWTYWAAGPLWGNYMFTLEPDHGQDRPQMEAIKPFLSQSAP

>*bglX*

MKLSRKIFLLSAVACGMMVAHDAAHAAHHAPGDPADEKARQVLAHMSPEDKMSLLFSVDGGGFNGSVAPPGGLGSAAYLRAPAGSGLPDLQISDAGLGIRNPAHIRKDGAAVSLPSGLSTASSWDMDMAREAGAMIGREAWQSGFNVLLGGGADLTRDPRGGRNFEYAGEDPLQTGRMVGSTIAGVQSQHVISTLKHYAMNDLETSRMTMSADIDPVAMRESDLLGFEIALETGHPGSVMCSYNRVNDLYACENPYLLNTTLKQDWHYPGFVMSDWGATHSSARAALAGLDQESAGDHADARPYFQTLLAADVKAGRVPQSRIDDMAQRIVRSLFAQGLVDHPSQRAPLDVVTDTLVAQRDEEEGAVLLRNEGNVLPLAPTARIAVIGGHADVGVISGGGSSQVDPIGGEVVKGPGKKDWPGDPVYFPSSPLKAMRAEAPAAHITYDPGTNIAAAVRAARAADVAVVYATQFTFEGMDAPNMHLDGNADALIAAVAAANPRTVVVMETGDPVLMPWNSSVAGVLEAWFPGSGGGTAIARLLFGKVAPSGHLTMTFPQAESQLAHPDIAGVTASNVFEMQFHTDQELVYDEGSDVGYRWFDRNHLKPLYPFGHGLTYTTFATDGLAAGYHHKQLTVTFNVRNTGTRPGVDVPQVYVGLPDGGARRLGGWQRVTLAPGESRQVSVQVDPRLLAHFDGRHDRWAIPSGRFRVWLGTSAVDDSQQVSIHLPARTLAP

>*bcsABII*

MATPRGLNWLILFLSAGGAFLCLSIGNITLDPGQQAYISVGTVALFFVLNRRKGRHITCILMMLSLFVSFRYLIWRLGSTVQFTGPLQIVMSVALLLAEGYALSTLCLSYFQMSWPLGRRPHSLPDNPADWPMVDVYVPSYNEDLDLVRSTVLGALDLHWPADKLNVYILDDGRRKSFYDFAQESGAGYIIRSENNHAKAGNLNHAMKITRGEYVVIFDCDHVPTRSFLLKTIGWMVADPRLALLQTPHHFYSPDPFQRNLAAGYDVPPEGNMFYGLVQDGNDFWDATFFCGSCAAIRRSALLSVGGFATETVTEDAHTALKMQRKGWGTAYLRQPLAGGLATERLILHIGQRVRWARGMLQIMRLDNPMLGPGLRWEQRLCYLSAMSHFLFAIPRVTFLVSPLGFLFFGQNIIAASPFAIMVYALPHIFHSIMTLSRIEGRWRYSFWSEIYETSLALFLVRITIVTLLQPHKGKFNVTDKGGLLARGYFDFSAVYPNVIMAGILFLGMVRGIVGMIFQYHQKLAFQSFLLNTFWISISLIVVLASIAVGRETRQIRHKPRVRVRLPVDICFEDGTEYHAHTTDISLGGAGVTLRLPRQMDTPQDITLRYDKPDDGIRVSVPATILGQRGSWLHLQWKIETLDQEREIVSLVFGRSDAWHNWADFKEDRPLNSIYQVIKSIRGLLSPPYLWDLSPAQETGESDESVQKEETLEKKSLVLPPTYRGVGYGMTLLVAGMAGLLSMVMPVQAQDTPAPAPVEAPAAAAPAVAAPARSLPPPSYSATGVSRLTPLGDTNSGDIPPVGSAVDRALADRVADTEITRTIPFRDLGLENGPLTLRGFSPLQGIDVVVPANRVVTHAVLTLSGAISPSLLPEATALTVTLNEQYIGTIRIDPAHPNFGPLELPVDPLYFTGDSKLNFHFAGEYRRDCNDLYNDVLWAQISDQSSITLTTARIVPERDLGRLPTPFFDPNLKVAMRVPVVMPAATPKPEIMQAGGLVASWFGRMADARQLSFPVSRSIPATGNAIEIGENIPVDAQGRPPAGPTLLEIANPNDRWGTVLVVTGRTAQEVEVAARALVFSTDTLGSGPSAVVQDVTLRARQPYDAPAFIPTDRPVRFGELVTAGDLQAGGFAPGTLHVPFHLPPDLYTWRGLPFLMDLWIRTPDNPVVDISASRLDVGLNNSYLQSYGLLPNSLWRTWSERMVTQHAGAVGHVTALPSWLLFGQNDLQFNFNTRPVDRGACRRTPDNLRMGIDSDSMLDFRRGVHFTTLPNLS

>*bcsB_IIa*

MADLGETTVVLPPAPDTDMIGAYLDLMGFFGSVTWYPTAGMHLVTTADIAEHPPSGDVILLAPAQQMGPAASLLSRSAYQIDDGHIHVGQDMGLQGIWYIFQDRDRSGLRNGVTANLNAPIRNTAMMIGAESPYARHRSVLALVGDDGAHIHDLIISLHDRKALPSLQGDLVLKNGNRFTTYRTSPTYTVGHLPFWMWVDWFLSRHPVLMYLCGVVGAAMLGTGAWLWLRARARRRVREQAIADEAARMAGHVSE

>*bcsABIII*

MVNKKSVYTGLLALVFVVFLLVSSLTYLPTDRQLFVAIGGVVLFFIVRRHHERWSRCFLMFLSIFVSTRYLVWRFTSTLDFSGVLQTIMVLVLALGEIYTTIRVGLTYFQLAWPLRRTVHPLPDDTSRWPVIDVYVPTYNEDLSIVRTTVLGCLALDWPADRLNVYILDDGRRAAFRDFAHQSGAGYITRMHNNHAKAGNLNHALEITTGDLIAIFDCDHVPVRSFLKKTIGWMVADPNLALLQTPHHFYSPDPFQRNMSRGRGIPPETNLFYGLLQDGNDYWNATFFCGSCAVLRRKAIMSINGFATETVTEDAHTALRMQRQGWGSAYLREPMAAGLETETLGMQIGQRMRWARGMFQMLRIDNPLTGPGLKLTQRICYFAAATHYLFSVSRLLFLLAPLGYLFLGVTMIAASPYELAVYALPHLFHTTMTMSRLQGRWRYSFWSELYETVLAPFLVRMTFITLIAPHKGKFNVTDKGGLLEHGYFDWRAVYPNIIIAMALVTGLGMGVWSACVHYHETLVFRAMAMNSLWILFSLTIVMGSIAVARETRQRRTCHRVQAKLPVHLATDDHQQFACHTRDVSMGGCCVSLHAADRFPVGHGVTVSWTLAGGPAAVRAVVIARTDSTVRLKWAIDGLAQEEQVVELVFGRDDAWAQWSDFPPDNPVRSFYTVLLSILALFRPSPRDGETKQAPETGPVVTDMEKEAVLPKERLVIQPTRRPRGAAAGIMALCLMLATGMTAWAQVPAAPAAVPTGAGTPVADRDGDNAVAVFDDEHMTQADAAHVSRTPVTVSRTFTQLGQFQPMLLRAMAPIYGMDADVGRDKIIRSARLSLSGSVSLSPTTGQAAIAVMLNDQPVGVVCTQGNGRFGPVDLPVSALLFDTRNRFNFRLIARQGAGATFPSGACGPDAQPPADGGMPMKATGLQVAIDPDSTLSFTTVQLVPHRLLSALPYPLVDRGMAGTAPVTFVLAPAASTRVQAAGGMVASWLGMKLHDRRIHFAVAPALSGPGNAIVVGVGLPGPWGNAPAGPVVAEIPNPHDVFGTVLVVSGRTDTDVATAARALVLGAPQLQIDGTHAAAPDVTPPARRPYDAPGLLPTDHPVRLGDLLPDTQLHRAGLDPMVVDIPFAVPPDLHTWRSKPFIARLRVMAPPGALVDRQHSEVDVMLNGMFLHSYPLVSWSFNPFGRHDGLVEHDVELPSWVLKEQNVLSVYFDVRARTSGGQGPAVSEVGLDPSSTIDLSASRHLAVLPSVKLFAMSGFPFSRMADMSQTTVLLPPDPAPATQGAFLDLMGFLGAVDQYPVTGLHVDTTDMHAGGEQAGDMLVISPFDRLGAASQALARAGYRTGGTWSVKAGQWMRHLVGMEAGNPPGSLDEGALIAAQSPFAPHHSVVALLGATPDALSAMAGDLRDPARGERFQGDMVVRHGARLDNYRTASAYTEGYMPAWMWPDWYLGGHPFLLYLLGVIGSVAGTSCAMSVLRARSRKRVLNDDLTGNL

>*bcsCII*

MLAGALGMLGTMGAARAAVPPSVSYAEGIVNQQIEEGYFWINHNEDTHALHALQRALQIQSDNIEALLMLGAVQVHQGDPAAARQTLLRLENAHGSPEQIAALQQWIAQPPVDRVALSHARATADAGHATQAMFQYRALFPQPLLSPMLEVEYDRVLSGAPLGYADAVRRLRTLLSIGPHDMEARIALDQSLGYRPVSRPEALADMRALATSPDTSSMIRDEAVQVWRRTLDWMGVDPQAAPYYRAWLDIHPDDTVISQRMQQIERMQRVNAGFTALAHDNLDQADAAFQDCVAEPSVRAAATEGLGLVAQRRGDMTKARALLTQAVALDPANVEIHRALAALDAPENDPALPQMWQLVSHHEYDRAWALLSEIEKRHGQIADTVQVRAIILEARHDWSAAKVAWTDLLRLSPGNPSAEAGLAGILIREGRLDEAARQIDRLRAVHFARLPVLEGLLLQARAQATTDPRLRATLLEQALRQLPDNGWDHLHLAQALVALGQTDRAQTLMQHFCDTAPRRTDALQACFAFAMQTHDMSGAAALLAHMPPAAITPDMARGVALVTLWKRLQALPASDAQAVAMLQDMPVAPDPDGMRGQMVVDAMLRRHASNAQAIAVLDRAMAQAGGTLGVDQALAYGGTFVQLGDPSAAQRVMDRLDAEGLDLTPQQRDARQQIRESIVVTRADQYDVAGRPDLAEKVIDPLLAQNPDSVALLLARGRVENVRHHSRQALDFDMRALHLKPDDRVVQATVARDALAVGQDHVARDMAASLMDHHPKWGDTWEIQAELDGHAGRERRQLGDLRHARMLDCATSGSLEEQMNSAMRLDPGCAEGRTRARDERPDASLPFIVGSGVAMPETYTYDPRLTPVQTLDRQAGYLSRALAPQADGNVEIRDRSGQSGLGHLTSINIPMTATIPLSSTRQTVSFSIMPSVLLSGNALAQPANAQQYGTVAANGVRPGFHMPGAVGGVAVGVRYKYDWITADVGSTPLGFTTSNVVGGIELAPQLTRNLTLRVTGERRAVIDSLLSYAGARDPGTGQVWGGVTRNRGHGQLEWGTQAYNVYAGGGYAVMKGTNTVANHEAEAGAGGSAQIWKGRDTQHLRLGLDLVYFGYKRNTYYFTWGQGGYFSPRAFMAALVPVTYDGHSGRWSWMFKGEAGYQHYTQDPTAVFPLGGRGVQGHQTYAGLSTGGLAGNVQARMIYQLTPGWRMGLEGGYSRSGSWDEVHGIFMIHYAPSH

>*bcsABIV*

MMLSLLVSLRYIVWRLTATVQFDNWLQTGLAVLLLLAEAYALMTLCLSYFQMAWPLRRKEHPLPDDMTQWPSVDVFVPSYNEELSLVRSTVLGALDLDWPADRLNVYILDDGRRKAFHDFAVAAGAGYIIRAENNHAKAGNLNHALAVTDSPFAVIFDCDHVPTRGFLRRTIGWMMADPRLALLQTPHHFYAPDPFQRNLAGGMHVPPEGNMFYGLVQDGNDFWDATFFCGSCAVIRREAVMGIGGFATETVTEDAHTALKMQRRGWGTAYLREPLAAGLATERLILHIGQRVRWARGMIQIMRLDNPMLGAGLRWEQRLCYLSAMSHFLFAIPRLTFLVSPLAFLFLGQNIIAASPLAISVYALPHIFHSVVTLSRIEGRWRYSFWSEIYETSLALFLVRITIVTLLQPHKGKFNVTDKGGLLARGYFDWDAVYPNVILAGVLCAALLRGVFGIVWQFHDRLALQSFILNTLWVVISLIIVLASIAVGRETRQTRNAPRVSVRLPVVVTDAHGRQMAGHTRDISLGGLAVGTRLAAPDMVGGTVTVRYDNARDGIHVGVPARVLEARDGALRLQWAVRDLADERQVVSMVFGRNDAWASWADFAPDRPLRSLAMVFRSIGGLLRRRPAEAPRALHEMGEGELPATEEKLEKQSFVLKPVPRSARHGATVSAALFVAFTALVPAAMAQEAPSPDQSGVSAETPFGDSNTGVVPDAPPAIDPAAADRISDAEVTRTLTFRNLGATTGPLTLRGYSPLQGLDVVVPANRVVTHAQLTLSGALSPSLLPEASAVTVTLNEQYVGTLKVDPQHPQFGPVSFDIDPLYFTGDNKLNFHFAGEYRRDCNDLFNEILWARISDMSRITLTTVRITPERRLSRLPAPFFDPNQRATLRVPVVLPATGDRGALRAAGLVASWFGRIADFRKLSFPVSTAIPASGNAVEVGVNLPVDAGGGRPAGPMLAEIANPNDRWGTVLVVTGRTPQEVEVAARALVFSPDTLGGVASKVVSDVSLGARHPYDAPAFVPTDRPVRFGELVGAADLQGGGFAPAGMTLPFHLPPDLYTWRGRPFLMNMWIRAPGGPVVDLETSRVDVSLNNNYLQSYTLSPPGLWRKWSERLVNQHAGAVGHVTALPPWLLFGQNQLQFNFDARPIDRGACRRTPGDIHMSVDSDSTLDFRRGYHFAEMPNLSYFAEAAFPFSRMADLSETTVVLPDHPDTGTTGAFLDLMGFFGASTWYPSAGVTIMGAGEVEQAPPKGDIVVLGTAAQLGAASGLLARSPYVIHDGRITVGQRMGLQGIWYLFQDRDHAGLKNGVTASLNAPIVDAGVLLAAQSPYDSQRSVVAFTGDTPERIHDLVLSLRNRSDLPSLQGDLVLKNGDQFTSYRTAPVYTVGSLPLWLRLDWFLGHHPSALYLAGLAGAGLAALGVWAWLRGWSRRRIARDDLTGEL

>*bcsX*

MNALLAGLTLLIIGDSHVTFKDSLLSVLPDEFTRQGAKVVTYGVCSSTAADWVVPNPNNGCGAAERVGDAPIGAPDMKPASPPPITSLIEKWHPNVVMVILGDTMAAYGQNAVSKDWVDEQVKTLTYAIGRTACIWVGPTWGQFSPRYGKTDQRATEMAGFLKGEVAPCSYVDGTALLKQGSVNTIDGIHATPESYRVWGDAIVQATLPELEKLKDAPPAPAQ

>*bcsY*

MLQLNPTPPAPSRWRAILENDFFPGNRRRDIDGLRGLAIALVVLFHAGWLKGGFIGVDVFVVISGYFMGRSALMQHPFQPVRFVCRRLYRLLPALLCMVALVSAGMLWWVLQSDRADIAINGAYALVYLSNIWASGHVGYFQGQAVAYPFLHTWSLSLEMQFYAIIFVMALLLPLTRHRRLVLSAIFSASAAYCAHAWYTGDSQAYYNIFDRLWQFALGTMVWMLPRPKLPAAAANAVYAAAVAVIVGAGLFYPLSSACPSWMTVFPCSAVVLIIMLPDTQVGRWCLVPLSPLGVISYSVYLWHWPGIVVANYLLFFQVHGAMMAGVLALVMVVSLLSYVLVERTGLDYEDRAPVAARNRGAALLVAACLGLAAVLAYISHVSRVH

>*bcsCIII*

MIRPRGPVPRDGAAWRRGPARWVLLCDAMRGREGGLLVACAVMAGLIASGGGAACAQDSHMAVAGAPAAPAAPTLPVTAADAADAAHLAHAAAVLELLLDQGYYWLGQHNLGKARETIQRALSIEPDNNEALFLQGRLQMGEGAAAQATRTLERLERQNAPAGLVADLKAQIQAGPVDPRALAEARALAASGRMMPAMFKYKALFRNGDPPPDLALEYYRVLGATILGYQEARTKLAAWVARNPRDIDARLCLDRILTYRVTSRAEGLDGLRVLARSSVSAQIRSDAVAAWRDALLWEPITGQSIPLYNEWLALHPDDTEFTIRLKKAQETQAGVDAANARQQGYALLARHMLDAAAREFHRAVEIDPHDPDALGGLGLVAQARQQPALARQYFLQAMQAGPDAAAHWHAALKALETGGGVDPLVARIVQAINDGRYDAARADLATLGHRPGYGLTVLSLQAALARRQGDTADAVRLYREVARRAPRDAGALFNLGALDVQVGDGTEAADILTRLQRLSPVMARRLEAMMLSAQADRAGDDDGRIVLLRRAQALDPDDPWVRLKLAHALDDAGDHAAAQAVMDALTAPRNASAQALQAGIIYAMGRHDTATASALLDRMPRTGRTPDMDRLASLVVLDQRIAALSHAPAAGNAAVLALADQPDPTGERGMRIAAALLARHAPQDARQALARGESLTQPPTPAQMLAYAGTYLRLRSAFDTTRCLDAFDAMAKARPADITAGQARAREQVAIGLAVMTADGFDRYGQTAQAAQVLAPVLRAHPDSVEAHLAMGRVYQARNMATRALDEDETALRLKPANIYALAAAARDAGGAHHLAQAKGYAARLAHEDPDGPMSWEVRSDIERIEGNSRGQLADVEHARHAQCTLDGEGECGGHESFVSDYRWPLIDSAYIDLHGATLPASYHYIPEDDGAQAMDRQIVYLRDSVSPQFDANTFVRSRTGVAGLGQLTEFAVPITATLPFESWDHRLSFSVTPTLLFTGDPLTSAVSAHQFGTVAVNGAQPWGYHHYYTQGVGLDLNYVNRWFAADVGSSPLGFPIANVVGGVEFAPRLTRNLGLRISGGRRMVTDSELSYAGERDPGTGKLWGGVTRLFGHGALEWSARGWNAYAGGGFAYLSGTNVIGNTETEAGAGGSATVWQDHDRQWLRVGLDLMYFGYRRNAYFFTWGQGGYFSPRQYFGAMVPVEWSGHNRRWTWFLRGEAGYQYYHSNAAPYFPTSAQLQDRADGNPPDCYGDSGASGLAGNMRGRLVYQVDHRLRIGLEGGYSRAGSWSETSGMWMAHYTLDGQ

Putative proteins involved in carbohydrate uptake and metabolism

> Carbohydrate-selective porin_I

MRRIWHNHHIWLHTIILLKTCSFAYAQPVPVSVLSAGVTPQQNIPDHIDPRTHLSGNWGGIRNWLLAQGIDIRISDTNEFWSNPVGGAQASSNYIGSAAVEMVTDLHTLTGLPLGTFDISAMEIRGRPFSNTPLYVFNQTSNIEADDNGRLYELWYSQKFMGERLAFRIGKLDLGHDFMVSSVGLNFLNASFSWPIMPDNDLYDQGPVSPVATPAIRLRYTLSPHWNFLFAAADDNPVGGPFINAKDPWNQNRDPGGTRFSFRTGALFFGEVQYRRTLYGRQGTYKLGGYFDTGRFPDQSDLRKNHKTNWAVYAIADQTLQHFGRITELDAFIRGNWTADTDRNQIVYAADAGLSLKGPFGRAGDMAGFGAGLGAASPYLAQADRRSGLPMQGTEYHLELTYQIPITPWFMLQPDIQGIISPSGGVLDNKGQHVHDEAIFGLHSSVTF

> Carbohydrate-selective porin_II

MRVPSGFFLLSYFYWCSDFVICRYGRTAMIVLVRTVVCLGLFLSVAVPARAADVDATRTTVVQDSKPHEYPGWVRRILGTKADTHKLPEVSNPTPYAETQPTTPFYETTPGFLNAPYGPPSFGPPFGTTHLLGDWGGVQPWLQNRGLYLAVDVYEDLAGNVAGGKKRDYTVAGQVGATLDVDWNRVLHSRAWTRDLWLHMLVVNGHGRNLSRLFGDNSNQVQQIYGSRGNVIAHLVWAYFEKGWLDRRVDLSVGWIPTGVFFQNSPWVCNFMNVWLCGGESPTKFLRGGRGWPSGNIGGVLRVMPIKQLYVMGGLFAVSPHNYNGGISGWSWAQDGLGKLSTQAEIGWIPAFGRDHLAGHYKLGAMYDNARYDDLYEDIHGNSWVLTGQPARRQSGQTSVWVIVDQMLVRRGAGEMNGLILGGYYAYASGQTSQINHSFMAVLMDTGALWHRPLDSVGIAFLWAGFSRSAILSQEAAAGRGLALPGGNFGVPYGIQGHETIYEAYYGFHIADGLSIKPDFQYVNHVGGTTVFRDAVVLSTAMNVAF

> Carbohydrate-selective porin_III

MVGMNTFNGSAMDYQGGVTGPSNNTRLSEFSYEQNLFNRIINIRLGRIAPTKYLTNLPTDCQFVSFVCGNPGGWSFNANQSHWPVATWGGMLTIRPATNYYIQTGAYADNSWGFTRTGFPFSGNWSEAHANGTFIPVEGGYSSTYETEAYPKKYVVGFYYDSHNFSDAYLNTAGKPLLTNGGTPMNDGAHTEVYGEFMQTVWKPNRKSHEDLQIFGGAYIGSSGHPTVNAYYTLGVLKHGLPRRPHDYMGLIGVASVFNQRVTAALDTAVQVNGGRGNFARSTEGVEFTYGAEVLPGVLFRPYIDAVFHPDQELYAYVPDPKIHYSVGGGAQIMIRFNEAFDLPEIKPL

> Carbohydrate-selective porin_IV

MHMVICASEAPANGLPNGSLGDDASYALRASAQHPSTTMTGDWGGTRLYLRNKGIDLAGAWTNETAGNVTGGDRHTAAQTNQLAAIMDIDAEKLLKWKGASFNFTFTTRWGSNLITRAGLYTLQQPQEVWGRGQTSRLTQLWYNQKLGDHVSFKIGRLPTGADFDNVPCLTMVNYFCGATTGNMDGNRWLNWPVSTWGGLVKYNDPRWYFLAGAYEQNARNLDNKFFLGYVHGATGVLLPFETGLRIHLGSHHYPGVYRIGGWVNTANAPDILLAANGQPAVTGGLAMLERGSSHGGYLWFQQQLTGTFSEKPGQDAVVQQGLTAYVTFTMVDKETSPVSSQVTGSLRYLGLPWRRHDALTLAFGTNHVNSRLATLDWYQAGRKGPRRDSEFAIETDYTLQITPWVYIEPNIQFWVNPGGYTQREMVTVIGMKTGLTF

> Carbohydrate-selective porin_V

MLFPDTEMPVRARSTARTGLQAAALALGCLLYGAMPAGAQVMDRDIGSAPSLLVSRPLPKHDSHKTWVPPFSQPEAVAPNPVSKWLRRRGINFLVDNTNEFAGAITPPTRGSTPGFSNYKQGASNAGQYAMQLDVDWDKLAGWHGFATHMITVGRYGTTANRMFGDWLNHASEDYGGGGNVVVHLVYAYGEETLLGGRLAIAAGRMSEISDFAASPLFCNFQNGSFCGRPKGITDTNYDAGYPAGTWGFRVRGRPARSVYVQTGLYPIEDGIYQVYQHRSGFKFNGANIIGYAAPIETAWEPVFRHGTLPGHYKFGAQLFSTPQNDNYLDVAGAPYALSGRKQRQHGASWSTWVMFDQRLLHYHDKDSGLTALSGAIYNDPRTSLRKYLVYAALINRGYFRARPYDTMGFALTYTRIADGVTMTEQDMIDTGNRSSLPNHATGVQRDTVVMEANYAIHVMPGVIFTPLFEYYIHPNAQSNLRDAALLGFKSHIQLM

> Carbohydrate-selective porin_VI

MKYVIDARMIRTFSRFFIPLSLLLLALFTLSETASAQAIPGEGQGVIGAQPSLNVNSRRNVPVNDPGSFFSAPYGNQHFFGDWAGLQPFLLKHGVHIEADVHEELAGNFRGGAKQGVTDAGQVGVEVDIDWHKLAGAPKNFWTHTMIVNGHGRNDSTDYIHDSLAGVQQIYGARGNVVAHLVYMYAEQSLFHNRLDISAGWIPVGSFFAASPLFCDFMNVSICGNPAPGKYVPGGRDWPSGNLGVVMRVMPTVDTYIMASMFAVSPHSYNGGISGWSWAQSGLGKFSTPVEFGWLPEFGRHHLAGHYKAGYGYDNSQYSDLYQDINGNSAVLTGQPFRKQSGVSTAWFQADQMFIRNGAGPTNGLIALAGFMYTSGKVSAMKYHAWAGMVETGAAWGRPLDTVGAMFHYFEMSRASVLQQESSVLAGTPFLSNQWGKAWGIQTHEDVYEVFYNIHTARGMSFQPDFQYINRPGGTTTYHDAAVMGLQFNCIL

> Phosphoenolpyruvate-protein phosphotransferase

MKTTERRPKQRPRAGEVRLEGQPVSSGVAIGYAAVAHEPAPPPVDMARSDCDPAHERTRLHDAISRSVAQLGRLHDRLALLPEDGQVEIGSLLEVYRRMLGPSRLRRGIEARLEQGMLAEAAVMQETEALAALMLAPPGRPAPQGEDAVAARRRAGEFREIGRRLVRNLSGTPYRSFSALPDGAILVAEQLRPADAALIDPSRIAAVVTEEGGSTGHTAILLRALGIPAVLAAHGLLAQVGRRTRLVVDGATGRVIVRPSSRTAAAARVEVASYARERQMLGRLRRLPARLSSGEVLTLQANLEIPAELPMVAQSGAAGIGLLRSEFLFMNTDTLPDEALQEAIYLPLIEAMAGDPVTIRVVDWGSEKNSDALSRVGIGNGGDANPALAVRGIRLLLQHRALLETQFAAILRAAHAGPVRVLLPMVSLLEELQAARDIYARVGRRLRRRGVKIPDPLPPLGIMIETPAAALMADVLAQEAEFLAIGTNDLTMYTLAADRVATDVAALYDPLHPAVLRLVRMTADAGLRQRRPVSVCGELASDPLAVPLLVGLGVRSFSMHASAVPRVKRAIRAAAMDDCRRMACRALEATDGQEVRAMLAQYAHTLQHPEDG

> Phosphocarrier protein

MAEAVAQPASHQADVEIVNQRGLHARAAAKFVTVAEKFDAGVDVAHAGMTVSGHSIMGLMMLGAGRGETVTITTSGPQGPQALRALTRLVGAGFDEDD

> PTS fructose transporter subunit IIA

MIGLVFVMHGVLGETLKGELEHVVGPQAQAAVFNVTATSLPGTCRTALQQAIDSVDSGQGVILLTDMFGSTPSNVAVSVLETDRVEVLAGVNMPMLVKLAQMRGHADMQECLNGAEAAGRRYISVASHLPAACLSGVGECVGEADSVAGHGG

>Bifunctional kinase/phosphorylase

MNELVQIHASCAARGAQGIMLCGPSGAGKSDLLLRLIDAGYDLVADDRVCMHAGWASAPPALAGLLEVRGIGIVRMAYRARVRVVAVARLVSPTDYPPRLPPAPKRDKVVGQPVFFLDPAQPSAVARIGLVLDCVAGDRQLLDERQM

> Quinoprotein glucose dehydrogenase

MNSLIRSAPLLVAAIAVCALTGLYLLGGGLWLCLIGGSFYYVVAGILLLVTAFLLFRRQASALTVYAVLLLGTMAWAVNEAGFDFWALAPRGDILVPIGIVLALPWVTRHLQPAGMAARVPLVGAIVAAVVVVGIALTGDPQDIAGNLPPVASDAPEPGDAHQMPDGDWQAYGRTQFGDRFSPLRQINADNVSRLKVAWTFRTGDLRGPNDPGEITDEVTPIKIRDTLYLCTPHQILFALDAKTGRQRWKFDPKLSYNPTFQHLTCRGVSYHEDKADTQAADGATAPVECARRIFLPTNDGQLFALDAETGARCASFGNNGVVNLQDGMPVKTLGFYEPTSPPVVTDTTVIVSGAVTDNYSTHEPSGVTRGFDVHTGALKWAFDPGNPDPNEMPSEHHTFVPNSPNSWITSSYDANLDLIYIPMGVQTPDIWGGNRGADAERYASSIVALNASTGKLVWSYQTVHHDLWDMDIPAQPSLVDIRNEQGEVIPTLYAPAKTGNIFVLDRRNGHLVVPAPERPVPQGAAPGDHLSPTQPFSQLTFRPSKLLTDADMWGGTMYDQLICRIMFHRLRYEGTFTPPSLQGTLVFPGNLGMFEWGGLAVDPVRQIAIANPIAIPFVSRLIPRGPNNPATPDRSLPSGSESGVQPQFGVPYGVDLHPFLSPLGLPCKQPAWGYMSGIDLRTNKIVWKHRNGTIRDSAPLPLPIKMGVPSLGGPLTTAGGVAFLTSTLDYYIRAYDVTNGRVLWQDRLPAGGQSTPMTYAVDGKQYIVTADGGHGSFGTKLGDYIVAYSLPDQN

> Aquaporin Z_I

MRLPHLHLPYLHIHVHDRPLAGDPHPHNPFHWKLYFCEAIATAILMILGLSAVILLTAPGSPFSWPLMHHPYIQTALCGLCFGLSGTAAAMTPFGKVSGAHINPSVTLAFSLAKRIGGVDALNYMIAQVIGAFLGTAVVYEAGRMIAWWGNMAVAVRYGATVPYGRISIWWAMWSEMFVTAALIAMLYWLAAHPKWKFITPWSGGLFFLIMNPVTAWLSGNSVNFARTLAPALFAGQWTGLWIYVVGPFAGASLAVMAIRMNLLGKLHLLEARLVNFGHHGRVPGLDDPHRKLNHPDDPDHVPPGAGPA

> Aquaporin Z_II

MTATRLPGSRPLEDRPLAGAPHPDHPFHWKLYGCELVATVALMICGIVSVTVLTTPYTAIGRALGPHPIVQTALCGLFFGLSGTVAAFTPFGRVSGAHVSPSVSLAFSLAGRLGVVDLCGYVAAQMAGACLATVLLAALGHVLPGWGQMVQASAYAATIPFSGVAIIWPLVTEVVLTALLVLMLCYLAGHPVLKWLTPWAGGLFFLFCNPVSAWLSGNSSNLARSFGPAIVAGQWESFWIYALGPFMGAALAIWLIRSRLLGRIEVEEARLVNFGHHGRIPSLLHPGRHREDNV

> Glycerol uptake facilitator

MSYNEINGKRRTLRPGQRARPFPTQKCSFKNMAGQENRHKADSPFPSNDNKRIKWVNMLKNRQFLGELISECIAVMIIVLIGDSVAAMYTLYDPSPYKLSYWGVSIVWGLGVTIAIYVTGSVSGTHANPAVSVALALYRGFPWRKVPAYCAAQVLGGILGAALVYTLYQPVIDHYNQLHGLTRADGGAAGVFFTHPGEFITPFHAFMDETILTALLVGGIFAITCEYNTVAPQANSSALIIGLLVASIGACSGYLEAWAINPARDFGPRLFCFLTGWGGSALPSPGNFWWVPIIGPLAGGVIGAGCYQFLIRPFIPRQGSPVIPPVP

> Glycerol kinase

MNKKNRILAIDQGTTSTRSIVFDRDITALSVSRIEFAQHYPNQGWVEHDPEEIWSNVMSTAREAIEKAGGPGAIAGIGITNQRETIVVWDRETGKPVHRAIVWQDRRTTSVCVRMHEEGHEPLVRERTGLLLDPYFSATKIAWILDNVEGARAKAEQGQLACGTIDSFLLWRLTGGRVHATDTTNAARTLLFNIHTCAWDDDLLALFNVPRAILPEVRTNSEIFGETESDLFGEPLKVAGMAGDQNAAMVGQACFRPGTAKATYGTGCFALLNTGTTPVESENRMLTTIAYRIGDETTYALEGSIFVAGAAIRWLRDGLNLITHASQTDDMATRVPHSHGVYMVPGFVGLGAPHWDPDARGLICGLTLDATAAHIARAALESVAYQTLDLMDAMHEDGGEELSALRVDGGMSVNDWFCQFLADMLRTPVERPKQVETTALGAAFLAGLATGVWSSIAELEGTWSRGQLFRPTMDKAQRDSMVAGWHVAVRRTLSSTVAA

> Glycerol-3-phosphate regulon repressor

MSAEERHHEIITLVRTQGYVSNEDLAQRLNVAVQTIRRDVNLLARRGLVARHHGGAGLASSVENIAYSERQVLNRRAKEAIGHLAARQIPDNSSLFVSIGTTTEAFAKALRRHKALRVITNNLHVATPLSAQTDFQVIVTGGLVRFYDGGITGSTASTFIEQYRTDFAVIGISGIEEDGTLLDFDADEISVAQAMMRNARRVYLLADQTKFGRRPMGRLGHLSHVHGFFTDRQPSERICALLREHDVDLHIA

>Glycerol dehydrogenase

MTHSARIAVIGAGAWGTALALQAARAGAEVSLWARTPATMSAGRVMPRLPGHALPPRITVSDVMPRQADLILLACPMQHLRTIARSVPPCAPLIACCKGVEEATGLLPLQVLGEMFPHSVLGVLSGPNFAHEVAAGLPAAAVLASADRTQARRLADLLTTPTFRLYASDDPTGVQVGGAAKNVVAIAAGATIGAKLGENARAALITRSIAELSRLSHALGGRPETLSGLAGIGDLLLTCTGAASRNYRLGLAIGQGTPAQQAADALEGVAEGRATAPALLLLARQHGVSTPVIATVASLLAGTIDMEDASQLLLSRPVGHEFA

> Dihydroxyacetone kinase

MKRFFNTRETIVTEALDGFLRSAAGSHLCRLDGYPDTCVVMQREPDRTQVSVISGGGSGHEPAHAGFVGRGMLTAAVCGALFASPCVDAIVAAILATTGDAGCLLVVKNYTGDRLNFGLAAERARALGKQVEMVIVGDDIALPDSATPRGVAGTVLAHKLAGYGAMQGWPLTRVAEFVRDAARRMRTIGLALEDCNPYEPDRASRLSADQAELGLGIHGEPGAQRIAMARADDLMRTAADTLEASLPTTVRNTRFALVLNNLGAVPEVEMALLLEAFSHTPLARRVSHVIGPAPLMTALDMNGFSITLIELDEAITTALQAAAQPRAWPGIAPLGSPAIAPMPPMPDAFPYPATPNPALRRVLERGAQVLVANEKALNELDGRIGDGDAGSTFAGAAREITAALDRLPLADPHHLMTTISNILTQHAGGSSGVLFAIMFSAAGRSPEPWRQALRDGLEHMMACGGAKPGDRTMIDALYPALEVLATGGGLPDVAHAAREGANSTTTMASARAGRAAYVPSAQVRDVPDPGAEAVARLLEGLAAG

> Oxaloacetate-decarboxylating malate dehydrogenase _I

MSQASTIRGQEILNNPARNKETAFTMTERQSLGLEGLLPPTVENIDRQLERVQTQLAAKPNDLERYIYLMSLAARNETLFYHTLMSDPARFVPIVYDPTVADACLAYGHIYRGSAGMYLTRDMKGRFKKILRNWPVKDVRFICVSTGGRILGLGDIGANGMGIPIGKLQLYTACAGVPPEALLPVLLDIGTTNAALRADPLYLGLRELPPDEAEVDLISEAFIEAATDVFPGVCIHFEDWKGTDAMRLLARYRDKVLCYNDDIQGTAAIAVAGLTTALQIKNETFADQRVLFLGAGSAGIGIASMIVSAMVEAGLGEAEARERIVLIDVNGLIETSRTDLNEWQKPFAHEAKPTKDLLEVVRTFKPTILIGVSTIGGAFTEEVVREMAKLNERPVIFPLSNPTNKAECTAEQAYTWTKGKALYAAGVQFPDFEYQGKSYHPGQANNFYIFPAIGLAVYATRPARITDDMFITAAAATADQVGPSAREHGMLFPLQANILETEVTTATRVAEHIFDSGQATVERPENIRAWIEGLLYKPVYKEL

> Oxaloacetate-decarboxylating malate dehydrogenase _II

MSHTSLKTALSGRELLDCPVLNKGNAFDRRERDLFGLHGLLPARVATLEEQVDLARARLAALPDNFSRHIALREIQDRNETLFYAIIDQALEAWLPIIYTPAIGRACREFSHIWTRPRGLFLTYADRGRIAEILSDPQWDGVRVIVASDGGSILGIGDQGANGMGIPIGKLSLYTACGGLDPARALPVLLDVGTDNTTLLEDPEYIGWRHERVRGTDYDAFIAEFVAAVNARWPNIALHWEDLSGADALRILRRYRDGMCTYNDDIQGTAGVTAGALLAAIRAGAAPLSDQRIVIFGAGGAGCGIADLLAQMMVADGMTPDEANRRFFMVDIDGLVRDGMDGVTEGQRPFVQPADIAATWNVADPAHVTLAEVMENVHPTMLIGTSGQGGAFTRDIVAPMVRQSVRPVIFALSNPTANIEATPADLLDWTEGRAIIGTGGPFAPVDHDGRSRPVDQINNSYVFPGVGLAVVAGGITRMTDGMFLAAAHALAGLSPAAGADDRTTAPLLPPVSELRTVAMAVARAVIRQGQVEGVAPQASPETLEAALKDAVWTACYRPYEKAQISR

> Pyruvate phosphate dikinase

MTKWVYSFGKGLNEGRADMRNLLGGKGANLAEMAANGLPVPPGFTITTEVCSAFYENGRKYPDALQAQVAEALHRIEQSMGLRFGDADAPLLVSVRSGARVSMPGMMDTVLNLGLNDETVEGLARSSGDARFAWDSYRRFIQMYGSVVMGVPHHHFEDTLEQFKRASRVEDDTAITADQWRAIVVDYRHIISTHAGSEFPTNPHDQLWGAIGAVFGSWMNPRANTYRKLHEIPASWGTAVNVQSMVFGNMGDDCATGVCFTRDPSTGENIFYGEYLINAQGEDVVAGIRTPQPMACARAEAGQHPMETTLPEAYGELLRVRSVLETHYKDMQDIEFTVQRNVLYILQTRNGKRTAAAALKIAIDMAREGLITQEEAIQRVPAASLDQLLHPTLDPKAERVQLTRGLPASPGAAAGAVVFTAEECEARAAKGEDVILVRIETSPEDVHGMHAARGVLTTRGGMTSHAAVVARGMGRVCVAGAGSIHVDYAAGTMTVGTHTVAQGEWITLDGGTGAVYLGRVPTIEPALSDDFNTLMGWADAVRRLGVRANAETPDDARTARRFGAEGIGLARTEHMFFGPDRIGFVRQMIIADDESVRQKAIAALLPFQRDDFASLFRIMAGLPVTVRLLDPPLHEFLPHAEAEMAEVANALGKSVDDVRARCAALAETNPMLGHRGCRLGLTSPEIYAMQVRALIQAAVMVEKELGKPIRPEIMIPLVATQAELATTRRAAEDEIARVLKEEGTNLNYYIGTMIELPRAALQADKIAEYADFFSFGTNDLTQTAFGLSRDDAGSFLPYYVDHGLLPRDPFVSIDRDGVGALVRLGVERGRQTCPDLKLGICGEHGGDPDSIAFFDEVGLDYVSCSPFRVPVARLAAAQAALATRQKAGQTA

> xylose-proton symporter

MMKESIKNAMFVRSDRHDVGYVLRICAIAALGGILFGYDTAVISGAVGSLQSYFHLSPAEIGWAVSNVLLGCILGAGVSGWLADRFGRRPTLAVSAALFTGSAIGAALATGFTSFVVYRFIGGIAVGVASSISPMYMSEVSPKDMRGRALYMESFAIVGGQLTVFIVNYLIARSASEAWLTNVGWRWMLGSEVIPCIVFCIAIFFMPESPRWHVLRGRDADAMRTLTRISNERHARSVLTEIKESLEHKIETSDLGRAVASKGARWIIFVGAMVAMLQQLTGVNSMMYYAPLVLGSVSGSVQNALFQTIWIGVASVSGAVLGSWVIDHKGRLPLFRFGSIGMIIGLLATSWALYTQTHGYSALVGMLVYMLLFGLSWGPLTWVLIAEIFPNRIRGVGMSIAVSANWVMNFIVSQLFPMMAQNHRLDALFHGALPMWLFALFTLFSWWFVERYVPETKGIALEKIESVMLTPKRRRAAAGTPTVPTDGLTDMVGRK

> xylose transporter

MAHDRQTASGSGIVNLIAGVAATGGLLFGYDTGIISAALLQITSDFGLGTAGQQVVTSAIVAGALGGCLVAAPLSDRLGRRYMIMFAALVFIVGTLVASFSPGVVILVCARFILGLAVGMCSQIVPVYIAEIAPREKRGQMVVLFQLAVVFGILVSFIAGYLCRHHSWRLMFGLGVVPAVILFVGMSVLPRSPRWLAMKGNMEGAFEVLRRLRSNPQAARAELDSIIAMHDEQAPWSALLQPWVRPAVVASVGIALFCQITGINAVLYYAPTIFAGVGFGESSALLTSIAIGVAMVISTAFGSWAVDAWGRRRLLLRLVPGAAVSLMVLAIMFGIGSTSGINTWITAAAVVSYAIFNVGSLSVAIWLVGAEVYPLSCRSKGMSLVAATHWTADLLISLTTLSLVQALGAAGTFWMYAALNLAAFVFVWRYVPETRGRSLEEIETALKAGTFNKLT

> xylulose kinase_I

MFVGIDLGTSALKAVLVDGQQNVIGSHSYPLRVSSPRPGWNEQAPQDWWMALLGAMDALAAAHPADMAQVTGMGLSGQQHGAVLLGRDGEVLRPCILWNDVRAVAQCAEFERRFPQSRDVCGNIAMPGFTAPKLLWVAEHEPEVFAATRHVLLPKAWLRYRMTGDMIEDMSDASGSLWLDVGRRRWSDDALVATGLRRDDMPDLVEGTARAGVLHAALAQRWGIRARPVLAGGAGDNAAGAVGLGAVRAGSSFLSLGTSGVLWVTTDRFRPHPQGGIHAFCHAVPDMWHQMGVTLSAASSLAWWARTTGMAEGDLLAELPARITHPSPAVFLPYLSGERTPHNDGHIRGMFAGLSHNTTRADMTQAVLEGVAFSFRDVVDALVDAGSPVTQADVIGGGSHSRAWVSILSSVTGLSLHRLAHGEQGGAFGAARLARIAVTGETVDAVCLPQQRKETLVPDPELASAYAAPLAFYRRLYPALRSAMDARSA

>xylulose kinase_II

MFVGIDLGTSALKAVLVDDAQQVVATSTRPLRISTPHPGWSEQAPQDWWAALIQAMDALAADHRGEMAAVTGIGLSGQQHGAVLLDAHGAVLRPCILWNDERAARECVEFERRFPRSRQVCGTIAMPGFTAPKLIWVARHEPDIFRATRHVLLPKAWLRYRLCGEMIEDMSDASGSLWLDVGQRRWCPDALAATGLSPAAMPALVDGTQRAGMLHQDLVGRWGMTARPVIAGSAGDNAAGAVGLGAVRPGSSFLSLGTSGVVWVTTDRFRPHPDGGVHAFCHTVPRTWHQMGVTLSAASSLAWWARMAGLPVADLLAELPPRVDRPSPVMFLPYLSGDRTPHDDPDIRGAFAGLSHDTTRADMTQAVLEGVAFSFRDVVDVMAQAGSDITQADVIGGGSRSLLWVSILSTITGLSLNRLAHGAVDGAFGAARLARIAVTGESVDTVCLPPQREATIHPDPALVEGYRTRLACYRALYPAIRRAFARPPSDHG

**References**

1. Gao, F. & Zhang, C.-T. Ori-Finder: A web-based system for finding oriC s in unannotated bacterial genomes. *BMC Bioinformatics* **9**, 79 (2008).

2. Seemann, T. Prokka: rapid prokaryotic genome annotation. *Bioinformatics* **30**, 2068–2069 (2014).

3. Benson, G. Tandem repeats finder: A program to analyze DNA sequences. *Nucleic Acids Res.* (1999) doi:10.1093/nar/27.2.573.
